# The ESX-5 System of Pathogenic Mycobacteria Is Involved In Capsule Integrity and Virulence through Its Substrate PPE10

**DOI:** 10.1371/journal.ppat.1005696

**Published:** 2016-06-09

**Authors:** Louis S. Ates, Aniek D. van der Woude, Jovanka Bestebroer, Gunny van Stempvoort, René J. P. Musters, Juan J. Garcia-Vallejo, Daisy I. Picavet, Robert van de Weerd, Massimiliano Maletta, Coenraad P. Kuijl, Nicole N. van der Wel, Wilbert Bitter

**Affiliations:** 1 Department of Medical Microbiology and Infection Prevention, VU University Medical Center, Amsterdam, the Netherlands; 2 Department of Molecular Microbiology, VU University, Amsterdam, the Netherlands; 3 Department of Physiology and Cardiology, VU University Medical Center, Amsterdam, the Netherlands; 4 Department of Molecular Cell Biology and Immunology, VU University Medical Center, Amsterdam, the Netherlands; 5 Department of Cell Biology and Histology, Academic Medical Center, University of Amsterdam, Amsterdam the Netherlands; 6 Eyen SE, Prague, Czech Republic; McGill UniversityHealth Centre, CANADA

## Abstract

Mycobacteria produce a capsule layer, which consists of glycan-like polysaccharides and a number of specific proteins. In this study, we show that, in slow-growing mycobacteria, the type VII secretion system ESX-5 plays a major role in the integrity and stability of the capsule. We have identified PPE10 as the ESX-5 substrate responsible for this effect. Mutants in *esx-5* and *ppe10* both have impaired capsule integrity as well as reduced surface hydrophobicity. Electron microscopy, immunoblot and flow cytometry analyses demonstrated reduced amounts of surface localized proteins and glycolipids, and morphological differences in the capsular layer. Since capsular proteins secreted by the ESX-1 system are important virulence factors, we tested the effect of the mutations that cause capsular defects on virulence mechanisms. Both *esx-5* and *ppe10* mutants of *Mycobacterium marinum* were shown to be impaired in ESX-1-dependent hemolysis. In agreement with this, the *ppe10* and *esx5* mutants showed reduced recruitment of ubiquitin in early macrophage infection and intermediate attenuation in zebrafish embryos. These results provide a pivotal role for the ESX-5 secretion system and its substrate PPE10, in the capsular integrity of pathogenic mycobacteria. These findings open up new roads for research on the mycobacterial capsule and its role in virulence and immune modulation.

## Author Summary

Mycobacteria are well protected from effectors of the immune system and from antibiotics by their cell envelope. The mycobacterial capsule constitutes the outer layer of this cell envelope. This capsule consists of glucan-like polysaccharides, proteins and glycolipid molecules and is thought to interact with the immune system of the host. In this study, we show that one of the protein secretion systems of slow-growing mycobacteria, called ESX-5, is important in maintaining the structure of this capsule. Furthermore, we identified PPE10, a protein secreted via ESX-5, as the main protein responsible for capsular integrity. We show that disturbed capsule integrity affects the ability of the mycobacteria to disrupt membranes. The bacterial mutants in *esx-5* or *ppe10* were also attenuated in a zebrafish model for tuberculosis, indicating that the capsular integrity is important for mycobacteria to establish disease. Knowledge on the mycobacterial capsule is only recently emerging and these results could open up new avenues in tuberculosis vaccine or treatment design.

## Introduction

Mycobacteria cause a wide range of diseases in humans, such as tuberculosis, Buruli ulcer and leprosy [1]. Mycobacteria are characterized by their unique mycolic acid-containing outer membrane (MOM). As the name implies, this outer membrane consists mainly of long-chain (C60-C90) fatty acids known as mycolic acids, which are partially covalently linked to the periplasmic peptidoglycan/arabinogalactan layer [2,3] and partially linked to trehalose molecules. In addition, this membrane also contains a number of unusual and specific (glyco)lipids. The MOM is extremely impermeable and thereby confers high intrinsic antibiotic resistance and provides protection against many harmful host factors. Although the MOM is in composition very different from the outer membrane of Gram-negative bacteria, electron-microscopy has shown that the form and thickness of the membranes are very similar [4,5]. Recent EM analysis also showed that there is a capsular layer surrounding the MOM [6,7]. This capsule is loosely attached to the cell-surface and consists of different (lipo)glycans, such as alpha-glucan and lipoarabinomannan (LAM) [8]. Growth with detergents, such as Tyloxapol or Tween-80, which are commonly used in mycobacterial research to prevent clumping, are known to disrupt the capsule [6]. Correspondingly, a recent study has shown that mycobacteria grown without detergents induce stronger and more diverse protective immune responses in mice [9]. The mycobacterial capsule therefore probably plays an important role in the interaction with the host, although the exact role of the capsule is difficult to determine, as there are no mutants identified yet with a complete loss of the capsule [10,11].

The capsule of *Mycobacterium marinum*, has also been shown to contain secreted proteins, mainly the substrates of the type-VII secretion (T7S) systems [6]. Pathogenic mycobacteria have up to five different T7S systems, named ESX-1 to ESX-5 [12]. Substrates of the ESX-1 system are required for the escape of mycobacteria from the host cell phagosome [13–15]. It is hypothesized that secreted ESX-1 substrates disrupt membrane integrity of the mycobacteria-containing vacuole [16–18] and thereby allow access of the bacteria to the cytosol [13–15,19]. Cytosolic mycobacteria are then able to proliferate and cause cell necrosis [13,20], allowing cell-to-cell spread of the bacteria. DNA released by cytosolic bacteria is recognized by the cGAS receptor leading to binding of ubiquitin followed by ubiquitin polymerization [21–23]. This leads to the sequestration of bacteria to autophagic vacuoles [19,23], which is an important process in the protection against mycobacterial infection.

Another T7S system is the ESX-5 system, which is only present in slow-growing species of mycobacteria. This system is involved in the secretion of the majority of PE and PPE proteins [24,25], which are named after a conserved motif in the N-termini [26]. PE proteins consist of a conserved domain of approximately 100 amino acids, which is necessary for secretion. In many cases this PE domain is fused to large C-terminal domains that are not involved in the secretion process. A large group of the *pe* genes (more than 60 in *Mycobacterium tuberculosis* and more than 130 in *M*. *marinum*) contain polymorphic GC-rich sequences (PGRS), which encode glycine-rich repeats that are postulated to play a role in immune evasion. Another group of ESX-5 substrates are the PPE-proteins, which are defined by a conserved N-terminal domain of approximately 180 amino acids that is required for secretion. Like the PE proteins, large C-terminal domains can be attached to this PPE domain [26]. Based on the paradigm substrates PE25 and PPE41 and the genomic co-localization of many *pe* and *ppe* genes [26], it has been suggested that PE and PPE proteins are secreted as folded dimers [27,28]. Although defined functions for a limited number of ESX-5 substrates have been described [29–31], most of the PE and PPE proteins have not been studied individually.

In this study, we show that the ESX-5 system is involved in the integrity and stability of the mycobacterial capsule. This effect is dependent on the ESX-5 substrate PPE10. Additionally, impairment of this process by genetic disruption of *esx5* or *ppe10* was associated with reduced ubiquitin association in cell infection and attenuated virulence in the early stages of infection.

## Results

### Identification and characterization of a transposon mutant in *ppe10*

**Fig 1:**
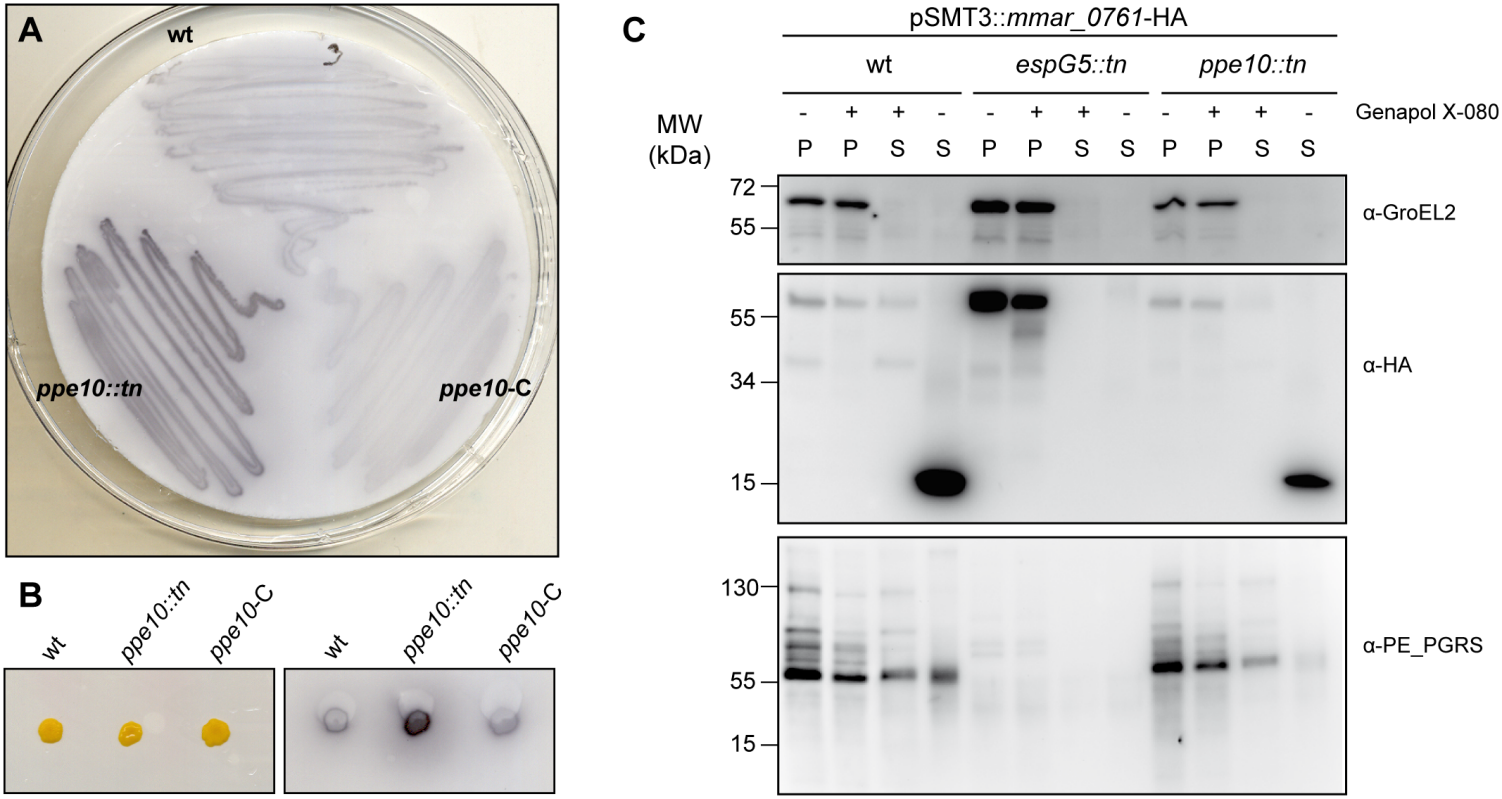
Identification of *ppe10*::*tn as* a PE_PGRS supersecretor mutant and its gene product as an ESX-5 substrate. A, B) Double filter analysis of wild-type *M*. *marinum* E11 (wt), the identified transposon mutant *ppe10*::*tn* and the complemented *ppe10*::*tn*-pSMT3::*mmar_0761* (*ppe10*-C) strain. The double filter of a bacterial plate culture (A), or of single colonies (B) was stained with a monoclonal antibody directed against the PGRS domain. C) PPE10 is secreted in an ESX-5 dependent manner. Immunoblot with protein preparations of wild-type *M*. *marinum* E11, the ESX-5 secretion mutant *espG*_*5*_::*tn* and the identified *ppe10*::*tn* mutant, all containing a plasmid encoding HA-tagged PPE10. The different protein preparations include cell pellets not treated with detergent Genapol X-080 (P -), cell pellets treated with Genapol X-080 (P +), Genapol X-080 supernatant fractions (S +) and culture filtrate fractions (S -). Depicted are the immunoblots stained with anti-HA antibodies or control antibodies (anti-GroEL as cell pellet marker and PE-PGRS as cell surface proteins). Full length PPE10-HA can be observed as a band with an apparent molecular weight of 58 kDa.

In an earlier study, set out to identify ESX-5 secretion mutants in *M*. *marinum* E11 [31], ~12,000 transposon mutants were screened for defective PE_PGRS secretion in a double-filter assay. In this assay, bacteria are grown on a nitrocellulose filter. Once visible colonies are present, this filter is place on top of a second filter. Proteins that are secreted by bacteria diffuse through the first filter and bind to this second filter. For this screen, we used a monoclonal antibody recognizing part of the PGRS repeat unit and therefore interacting with many (putatively all) PE_PGRS proteins [24]. The identified secretion negative mutants were described previously [31], but we also identified eighteen transposon mutants that secreted more PE_PGRS proteins as compared to the wild-type (i.e. supersecretion). Most of the identified transposon insertions were localized upstream of *pe_pgrs*-genes and were therefore not studied further, because the supersecretion phenotype was likely due to overexpression of those specific *pe_pgrs* genes. However, three of the mutants had a transposon insertion in the *ppe10* gene (*mmar_0761*), which is not next to a *pe_pgrs*-gene. PPE10 was earlier identified to be a substrate of the ESX-5 system [24,25], without any described function. Repeated double filter analysis of the *M*. *marinum-ppe10*::*tn* strain confirmed the supersecretion phenotype of PE_PGRS proteins. Importantly, this phenotype could be complemented to wild-type levels when the *ppe10* (*mmar_0761*) gene from *M*. *marinum* was reintroduced under control of the *hsp60* promoter using plasmid pSMT3::*mmar_0761* (Fig 1A & 1B). In addition to the secretion phenotype, *M*. *marinum-ppe10*::*tn* also exhibited an altered colony morphology (Fig 1B), indicating that the cell-wall composition could be altered. The mutant had a “smooth” colony morphology, similar to what was found previously in an *espG*_*5*_::*tn* mutant in the same background strain [24]. This colony morphology was also reverted upon complementation and the complemented strains showed a more pronounced rough colony morphology as compared to wild-type *M*. *marinum*.

The PE_PGRS supersecretion phenotype of *ppe10*::*tn* and complementation of the mutant was further confirmed by immunoblot analysis of culture filtrate fractions (CF in S1 Fig). As a control, we also included the pellet fraction and a fraction enriched in capsular proteins by treating whole cells with 0.5% of the mild detergent Genapol X-080, as described previously [6]. Typically, a substantial amount of PE_PGRS proteins ends up in this capsule-enriched fraction of *M*. *marinum* [6,25]. Multiple PE_PGRS proteins were present in higher amount in the culture filtrate of *ppe10*::*tn*, which indicates that this mutation has a general effect on PE_PGRS proteins. The PE_PGRS supersecretion phenotype of *ppe10*::*tn* was completely reverted upon introduction of the complementation plasmid pSMT3::*mmar_0761*, or the plasmid pSMT3::*mmar_0761*-HA containing an HA-tagged version of PPE10 (S1 Fig). As a control for secretion analysis, we included both the cellular protein GroEL2 and the ESX-1 substrate EspE, which is the main capsule protein of *M*. *marinum* [6]. The GroEL2 control protein was only seen in the pellet fraction, as expected. Surprisingly however, the capsular localization of EspE was completely lost in the *ppe10*::*tn* strain. This effect could partially be complemented by the introduction of pSMT3::*mmar_0761* (S1 Fig). In conclusion, the *ppe10* mutation seemed to affect both ESX-1 and ESX-5 substrates of the capsule layer.

Previous proteomic analyses by our group indicated that PPE10 is secreted to the cell-surface of *M*. *marinum* in an ESX-5 dependent manner [25]. To examine this in more detail we studied the behavior of HA-tagged PPE10 (PPE10-HA) in a wild-type strain, an ESX-5 secretion mutant (*espG*_*5*_::*tn*) [24] and the *ppe10*::*tn* mutant (Fig 1C). This analysis showed that a processed form of PPE10-HA of approximately 15 kDa is secreted to the culture filtrate of both wild-type bacteria and *ppe10*::*tn*. As expected, in the ESX-5-deficient strain *espG*_*5*_::*tn* this 15kDa product was not observed in the supernatant and full-length PPE10-HA accumulated in the pellet fractions, which confirms the ESX-5 dependent secretion of this protein. Surprisingly, only relatively low amounts of PPE10-HA were detected in the Genapol X-080 fraction of wild-type bacteria, although previous proteomic analysis did show the presence of substantial amounts of PPE10 in the capsule enriched fraction [25]. Therefore, perhaps only the C-terminus, containing the HA-tag, is cleaved and shed to the culture filtrate. The C-terminal cleavage of *M*. *marinum* PPE10 upon secretion corresponds to what was previously observed for *M*. *tuberculosis* PPE10 (Rv0442c) [24].

### Surface localization of capsular proteins is impaired in *esx-5* and *ppe10* mutants

**Fig 2:**
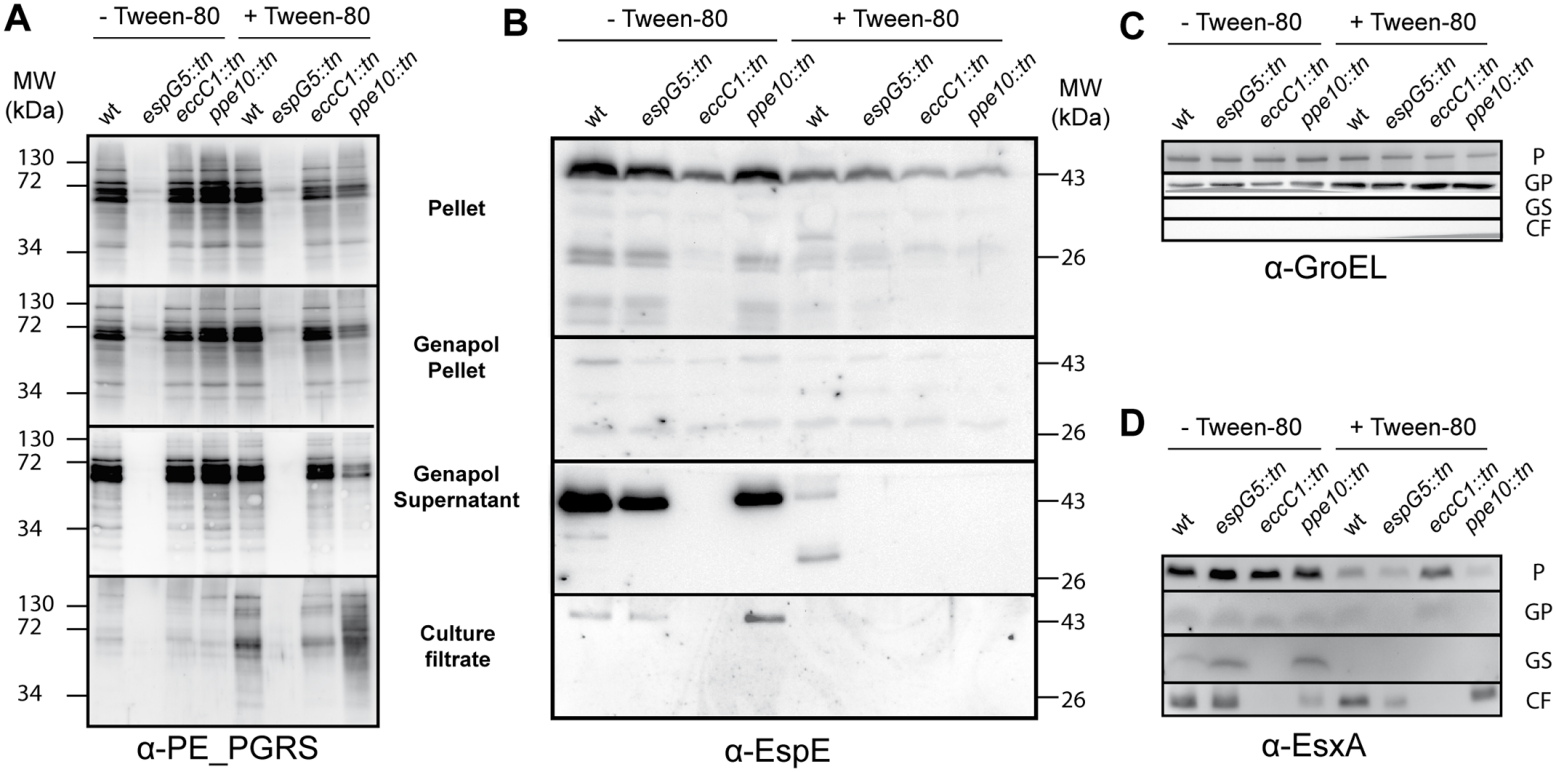
Both the *esx-5* and *ppe10* mutants release more capsular proteins when grown with Tween-80. Wild-type *M*. *marinum* E11 (wt), and isogenic transposon mutants *espG*_*5*_::*tn*, *eccCb*_*1*_::*tn* and *ppe10*::*tn* were grown overnight in 7H9 medium in the presence or absence of the detergent Tween-80. Cells (P) were separated from culture filtrate (CF), after which pelleted cells were treated with Genapol X-080 to enrich for capsular proteins (Genapol supernatant (GS)). A) Immunoblot analysis with α-PE_PGRS antibody confirmed the PE_PGRS supersecretion phenotype of the *ppe10*::*tn* strain, when cells were grown in the presence of Tween-80. When grown without detergent, there is no visible phenotype of PE_PGRS secretion in the *ppe10*::*tn* strain. B) The ESX-1 substrate EspE is present in low amounts in the capsular fraction of wild-type *M*. *marinum* when grown with detergent, but this residual capsular EspE is lost in both the *espG*_*5*_::*tn* or *ppe10*::*tn* mutant strains. C) α-GroEL was used as a loading and lysis control for all samples. D) The ESX-1 substrate EsxA could be detected in the capsular layer of cells grown without Tween-80, but seemed to be enriched in the capsule of *espG*_*5*_::*tn* or *ppe10*::*tn*.

Since the surface localization of both the ESX-1 substrate EspE and ESX-5 dependent PE_PGRS substrates seemed to be affected by the transposon insertion in *ppe10*, we investigated the capsule layer of various mutant strains, when grown in the presence or absence of the detergent Tween-80. Detergents such as Tween-80 or Tyloxapol are generally used in the liquid cultures of mycobacteria to avoid bacterial clumping, as in the experiments described above. However, it was recently demonstrated that the presence of these mild detergents also disrupts the capsule layer of mycobacteria and thereby alter the localization of capsular protein and capsular (lipo)polysaccharides [6]. For instance, upon culturing with detergent, the PE_PGRS proteins are present in increased amounts in the supernatant. The capsular protein EspE is also strongly reduced at the cell surface upon growth in the presence of detergents, but this protein does not accumulate in the supernatant, possibly due to proteolytic degradation [6, Fig 2]. When secretion of PE-PGRS proteins of *M*. *marinum-ppe10*::*tn* was analyzed by immunoblotting, it was evident that more PE_PGRS proteins were present in the culture filtrate of the mutant as compared to the wild-type (Fig 2A). However, this PE_PGRS supersecretion phenotype was only observed when *ppe10*::*tn* was grown with the detergent Tween-80. In cultures grown without Tween-80, the majority of PE_PGRS proteins remained surface localized, similar to the wild-type strain. These data therefore indicate that *ppe10*::*tn* does not produce more PE_PGRS protein, but instead this strain releases more PE_PGRS proteins from its surface, when grown in the presence of detergent. To analyze whether this was a more general phenotype, the localization of EspE was analyzed in the same secretion experiment. Western blot analysis confirmed that EspE is indeed localized in the capsule-enriched (i.e. Genapol supernatant) fraction, when bacteria were grown without Tween-80 (Fig 2B). However, lower amounts of EspE were present in the capsule-enriched fraction of the *ppe10*::*tn* and *espG*_*5*_::*tn* mutants as compared to wild-type *M*. *marinum*. Similarly, when *M*. *marinum* was grown in medium containing Tween-80, lower amounts of EspE were detected in the capsule fraction (Fig 2B & S1 Fig). These data indicate that also the EspE capsular protein is indeed more loosely attached to the cell surface of the *esx-5* and *ppe10*-mutants. We also tested the localization of the well-known ESX-1 substrate, i.e. EsxA (ESAT-6), since this protein is often implicated in mycobacterial virulence. There was no clear difference in the culture filtrate levels of this protein in the wild-type and the *ppe10*-mutant strain (Fig 2D).

### Capsule integrity is impaired in ESX-5 mutants of pathogenic mycobacteria

**Fig 3:**
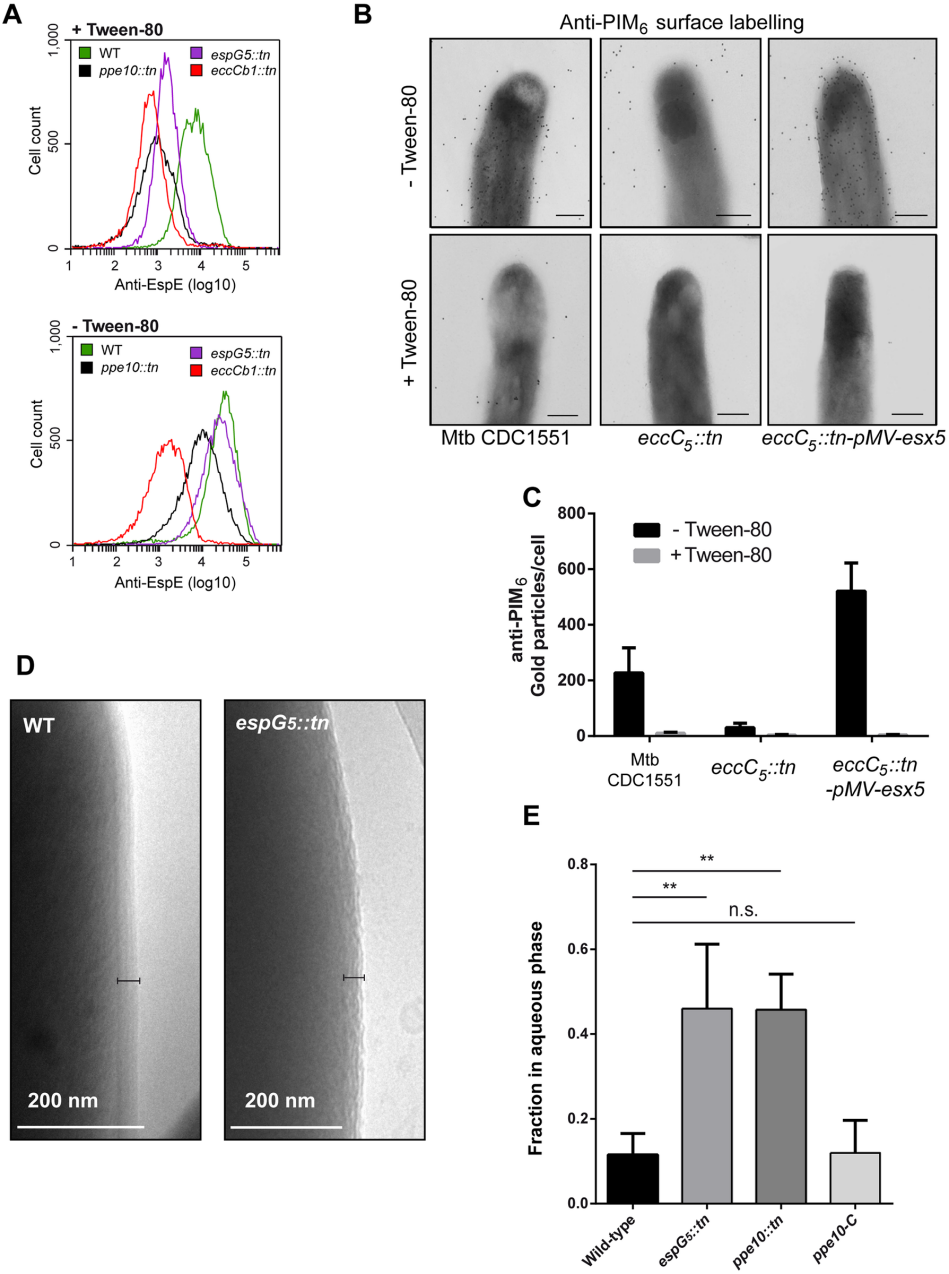
Surface labelling of capsular protein EspE and capsular glycans is dependent on ESX-5. A) Parental strains of wild-type *M*. *marinum* E11, or isogenic mutant strains *espG*_*5*_::*tn*, *eccCb*_*1*_::*tn* and *ppe10*::*tn* expressing pSMT3::*mCherry*, were grown in the presence or absence of Tween-80 and labeled with the α-EspE antibody and a FITC labeled secondary antibody. Subsequently, cells were analyzed by flow cytometry analysis. High levels of surface expression of EspE could be detected in the absence of Tween-80 in both wild-type *M*. *marinum* and the *espG*_*5*_::*tn* mutant strain, while *ppe10*::*tn* showed an intermediate EspE surface labelling. When grown in the presence of Tween-80, surface labeling of EspE was almost completely lost and reduced to similar levels of the ESX-1 mutant strain *eccCb*_*1*_::*tn*. B) Immuno-electron microscopy analysis of *M*. *tuberculosis* CDC1551, the *esx-5* mutant Mtb-*eccC*_*5*_::*tn* and the mutant complemented with an integrative plasmid containing the complete *esx-5* region; pMV-*esx-5*. Cells were grown in liquid culture with 0.05% Tween-80 (lower row) or without Tween-80 (upper row) and labeled with a monoclonal antibody directed against PIM_6_-LAM capsular glycolipids and a gold-labeled secondary antibody. C) Gold labeling of the different Mtb strains depicted in B was quantified by counting the number of gold particles per cell after growth in the presence or absence of Tween-80 (error bars indicate the standard deviation). D) The capsule morphology of wild-type *M*. *marinum* and *espG*_*5*_::*tn* was analyzed after plunge freezing of bacteria grown in the absence of Tween-80 by cryo-electron microscopy. The black bar indicates the mycobacterial capsular layer. E) Reduced hydrophobicity of the *espG*_*5*_::*tn* and *ppe10*::*tn* strains, measured as the OD of the aqueous phase after 1 OD of bacteria was incubated with 0.5% (v/v) Xylene in PBS for two hours. Statistical differences were calculated by GraphPad Prism software using one-way ANOVA and (Dunnett’s) multiple comparisons against a single control. ** = *p*<0.01, n.s = not significant.

To confirm a differential localization of capsular proteins in *esx-5* and *ppe10* mutant strains, we incubated intact bacteria with an anti-EspE antibody. These antibodies were subsequently visualized using a FITC-labeled secondary antibody, followed by flow cytometric analysis. Wild-type *M*. *marinum* E11 showed high levels of EspE surface labeling (Fig 3A), whereas the negative control strain mutated in the ESX-1 secretion system (*eccCb*_*1*_::*tn*) had only residual staining. As expected, surface labeling of the wild-type cells was reduced when the cells were grown in the presence of Tween-80, although the levels were still significantly higher than the negative control strain. The *espG*_*5*_::*tn* mutant showed EspE surface labeling similar to the wild-type bacteria when cultured without Tween-80. However, the presence of Tween-80 reduced EspE surface labeling to values only marginally higher than the ESX-1 mutant. The loss of EspE surface labeling in the *ppe10*::*tn* mutant was even more pronounced, showing intermediate EspE surface labeling in the absence of Tween-80, while EspE surface labeling was almost completely abolished when Tween-80 was present. Another striking observation in the flow cytometry analysis was that both the *espG*_*5*_::*tn* and *ppe10*::*tn* mutant showed an overall smaller particle size (S2A Fig). Calibration beads were used to estimate particle size of analyzed bacteria and showed that a majority of the particles in E11 wild-type cultures grown in the presence of Tween-80 were similar or smaller in size then the 1μm calibration beads (S2B Fig), which could represent single-cell particles. However, when E11 was grown in the absence of Tween-80, much larger particles could be observed, ranging in sizes up to 15μm. This confirms the formation of clumps of multiple mycobacterial cells under normal growth conditions without detergent. In contrast, *ppe10*::*tn* did not show any bacterial clumping in the presence or absence of Tween-80, indicating that the mutations causing deficiency in the mycobacterial capsule also affect the cellular aggregation of *M*. *marinum*. This behavior seems to correlate with the smooth colony morphology of this mutant. The ESX-1 mutant showed a more clumpy phenotype as compared to wild-type *M*. *marinum* (S2A Fig), with approximately 30% of the particles being larger than single cells even in the presence of detergent (S2C Fig). This shows that absence of capsular ESX-1 substrates is not responsible for the single-cell phenotype.

Tween-80 dependent surface localization of EspE in the ESX-5 mutant *espG*_*5*_::*tn* was further confirmed by surface immunolabeling of whole *M*. *marinum* cells followed by transmission electron microscopy. First the EspE antibody specificity was determined by analyzing *M*. *marinum* strain M^usa^ wild-type and *M*. *marinum* ΔRD1 (Δ*mmar_3871–9* [32]) (S3A Fig). As expected [6], EspE surface labelling of the ESX1 secretion mutant was significantly reduced as compared to the *M*. *marinum* wild-type strain (S3B Fig). EspE surface labeling of the ESX-5-deficient strain *espG*_*5*_::*tn* was completely dependent on Tween-80, *i*.*e*. without this detergent surface labeling reached wild-type levels, while surface labeling was completely lost in the presence of Tween-80 (S3B Fig). This confirms that the capsule layer of *M*. *marinum* is more loosely attached to the cell surface when the ESX-5 secretion system is not functional.

The effect of ESX-5 on capsular integrity was also examined for another mycobacterial pathogen, *M*. *tuberculosis*. As *M*. *tuberculosis* EspE is not recognized by our antibodies [6], we used antibodies directed against two different glycolipid components of the *M*. *tuberculosis* capsule, i.e. an anti-PIM6 antibody [33] and an anti-ManLAM antibody [34]. Wild-type *M*. *tuberculosis* CDC1551, an isogenic *eccC*_*5*_::*tn* transposon mutant and the *eccC*_*5*_::*tn* strain complemented with the complete *esx-5* locus [35], were grown in the presence of Tween-80 after which a sample was taken and fixed. Subsequently, the strains were grown in 7H9 medium without Tween-80 for six days and both samples were analyzed by immunogold labeling and electron microscopy. As described before [6], efficient surface labeling of wild-type *M*. *tuberculosis* was observed with these antibodies, which was confirmed in our experiments (Fig 3B, upper frame and S3D Fig, upper frame). However, when these strains were grown in the presence of Tween-80, surface labeling was strongly reduced (Fig 3B & 3C; S3C & S3D Fig). Even in the absence of Tween-80, surface labeling with both antibodies was completely abrogated in the *eccC*_*5*_::*tn* strain (Fig 3B, middle frame; S3D Fig, second frame). Complementation of *eccC*_*5*_::*tn*, by introduction of the *esx5*-locus on an integrative plasmid, resulted in an increase in surface labeling with anti-PIM_6_ compared to the wild-type strain. Together, these data confirm that the ESX-5 system also plays an important role in the capsular integrity of *M*. *tuberculosis*.

Next, we analyzed the morphology of the capsule of wild-type *M*. *marinum* and *espG*_*5*_::*tn* by cryo-electron microscopy. The mycobacterial capsule can be visualized as a defined electron-dense layer around the outer membrane [6]. This was clearly visible in wild-type *M*. *marinum* grown without detergent (Fig 3D, left frame). A capsular layer was also present in *espG*_*5*_::*tn* (Fig 3D, right frame), but with a clearly different morphology. The capsule of the *espG*_*5*_::*tn* mutant was 32 ± 3 nm (measured at 5 sides on 12 bacteria), which is slightly thinner than the capsular layer measured for the wild-type (38,80. ± 1,27 [6]). However, unlike the defined smooth structure of the *M*. *marinum* wild-type (Fig 3D, left panel), the *espG*_*5*_::*tn* mutant showed ruffled, uneven structures, which could be linked to the instability of the capsular layer under these conditions (Fig 3D, right panel). Together, these data demonstrate that the capsule integrity of pathogenic mycobacteria is dependent on a functional ESX-5 system. Our data obtained in *M*. *marinum* suggest that the ESX-5 substrate PPE10 plays a large role in this phenomenon. Although different components of the mycobacterial capsule have a different detergent extraction behavior, all tested components are more loosely attached in *esx-5* and *ppe10* mutant strains as compared to wild-type bacteria.

### Mutants in *esx-5* and *ppe10* exhibit reduced cell-surface hydrophobicity

Previously, we have shown that an *esx-5* mutant with a disrupted *eccA*_*5*_ gene shows reduced cell-surface hydrophobicity as compared to wild-type *M*. *marinum* [36]. Therefore, we also performed xylene extraction experiments with the *ppe10*::*tn* and *espG*_*5*_::*tn* strains. These experiments showed a significant (P<0.01) increase of both these mutants in the water layer, indicating increased surface hydrophilicity (Fig 3E). Importantly, this effect was fully complemented by introducing an intact copy of the disrupted *ppe10* gene. This reduced cell-surface hydrophobicity could explain the phenotypes of reduced capsular integrity and cellular aggregation of the *esx5* and *ppe10* mutants. We hypothesized that differences in cell-surface hydrophobicity could be caused by altered lipid profiles. To test this, apolar lipids, polar lipids and mycolic acids were extracted from wild-type *M*. *marinum*, *ppe10*::*tn*, *espG*_*5*_::*tn* and the complemented *ppe10*::*tn* strain and were analyzed by thin-layer chromatography (TLC). No significant differences for any of the lipids tested were observed (S4 and S5 Figs), suggesting that ESX-5 and PPE10 are not directly involved in the metabolism of the investigated lipids.

### Reduced capsule integrity of *esx-5* and *ppe10* mutants correlates with defects in membrane disruption and reduced ubiquitin co-localization in early infection

**Fig 4:**
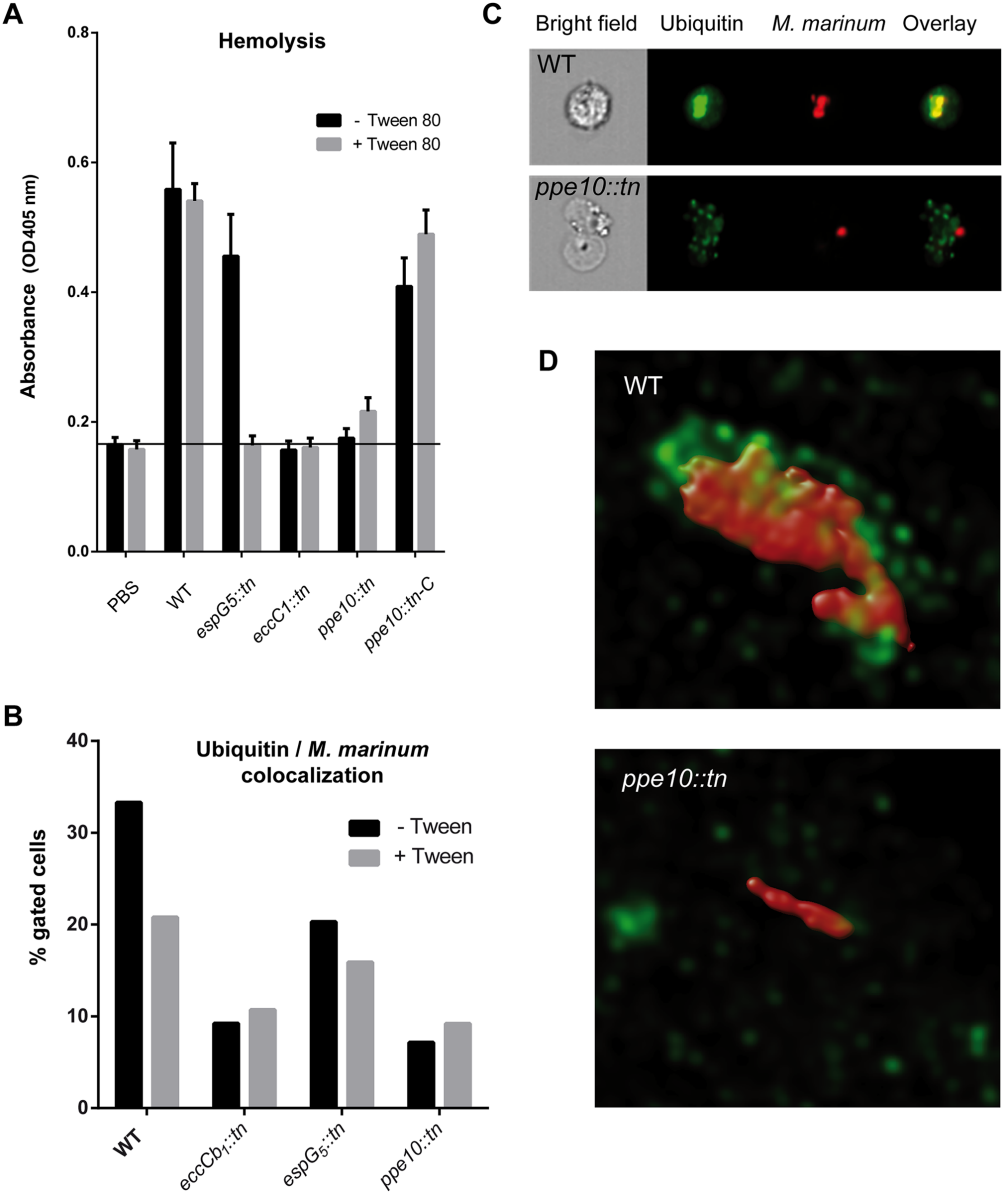
Both the *esx-5* and *ppe10* mutants have reduced hemolytic activity and show reduced ubiquitin co-localization upon infection of host cells. A) Contact-dependent hemolysis of red blood cells (RBCs) by *M*. *marinum*. *M*. marinum E11 wild-type cells and isogenic transposon mutants disrupted in *espG*_*5*_, *eccCb*_*1*_ and *ppe10* and the complemented *ppe10* mutant were grown in the presence or absence of Tween-80. Subsequently, washed cells were used for the hemolysis assay. B) Quantification of differentiated THP-1 cells that were infected with *M*. *marinum*. Only cells gated as positive for co-localization of the bacteria with ubiquitin were chosen for further analysis (full data and statistics in S6 Fig). The *ppe10*::*tn* mutant showed similar levels of ubiquitin co-localization as *eccCb*_*1*_::*tn* indicating that this mutant has no cytosolic access in the early stages of infection. C) Images obtained by imaging flow cytometry of wild-type *M*. *marinum* and *ppe10*::*tn* three hours after infection of differentiated THP-1 cells. Bacteria express *mCherry* (Red), while ubiquitin is visualized by FK-2 antibody against poly-ubiquitin (Green). D) Super-resolution confocal microscopy images of wild-type *M*. *marinum* and *ppe10*::*tn* illustrating differential bacterial clustering and ubiquitin labeling of bacteria. Bacteria express *mCherry* (Red), while ubiquitin is visualized by FK-2 antibody against poly-ubiquitin (Green). A 3-dimensional view of these images can be found in supplementary S1 and S2 Movies.

Because the *esx-5* and *ppe10* mutants have a reduced capsule integrity, we hypothesized that this might affect the virulence of mycobacteria. For instance, reduced capsule integrity results in lower amounts of surface localized ESX-1 substrates, which could affect the membrane-disrupting capacities of these mutants [13,37,38]. Therefore, the membrane-disrupting potential of different strains was tested by quantifying the contact-dependent lysis of erythrocytes in a hemolysis assay [37,38]. In line with published data, hemolytic activity was observed for the wild-type strain, independent of the presence of detergent in the culture medium (Fig 4A). As expected, the ESX-1-mutant strain *eccCb*_*1*_::*tn*, grown either with or without detergent, did not show hemolytic activity. In line with our hypothesis, hemolytic activity of the *espG*_*5*_::*tn* mutant was only observed for cells grown without detergent, while hemolytic activity was completely abrogated when the cells had been grown in the presence of Tween-80. This effect was even stronger for the *ppe10*::*tn* strain. Again, this effect was reversed upon complementation. These experiments show that there is a clear correlation between the PPE10-dependent surface localization of ESX-1 substrates and the hemolytic potential of the mycobacterial strains.

Next, we assessed the subcellular behavior of these mutants and their potential to rupture the phagosomal membrane. Different *M*. *marinum* strains expressing pSMT3::*mCherry* [39] were cultured overnight in the presence or absence of Tween-80 and subsequently used to infect differentiated THP-1 cells. After two hours of infection and one hour of incubation, the cells were fixed and stained with the FK2 antibody, which recognizes mono- and poly-ubiquitinylated proteins but not free ubiquitin. As ubiquitin is known to accumulate around cytosolic bacteria [13], ubiquitinylation can be used as a marker for cytosolic contact of bacteria [19,40,41]. Co-localization of bound ubiquitin and fluorescent mycobacteria was quantified using imaging flow cytometry (complete data in S6 Fig). For this, first the infected cells were selected and subsequently, the relative co-localization of ubiquitin with the intracellular bacteria was determined. When wild-type bacteria were grown without Tween-80, 33.3% of the detected bacteria co-localized with ubiquitin, while the amount of co-localization was only 9.2% for the negative control strain *eccCb*_*1*_::*tn* that is unable to secrete ESX-1 substrates (Fig 4B). Intermediate co-localization with ubiquitin was seen for *espG*_*5*_::*tn* (20.3%), while *ppe10*::*tn* (7.15%) showed comparable levels to the negative control. When the strains were pre-cultured with Tween-80, reduced co-localization of ubiquitin and bacteria was observed for wild-type *M*. *marinum* (22.7%) and *espG*_*5*_::*tn* (15.9%). Values for *eccCb*_*1*_::*tn* (10.7%) and *ppe10*::*tn* (9.19%) remained similar, indicating that these values are likely the background level of this assay. Representative images of these analyses are shown in Fig 4C, where it is illustrated how co-localization of bacteria and ubiquitin is indeed reduced in the *ppe10*::*tn* mutant strain (Fig 4C). High resolution confocal microscopy was used to illustrate this phenomenon in more detail for the wild-type *M*. *marinum* and *ppe10*::*tn* strains pre-cultured without Tween-80 (Fig 4D). Extensive surface labeling of bacteria by FK2 could be observed for wild-type *M*. *marinum* (Fig 4D, upper panel; S1 Movie), but was generally not present for *ppe10*::*tn* cells (Fig 4D, lower panel; S2 Movie).

Another observation from both imaging flow cytometry and confocal microscopy analyses was that *ppe10*::*tn* bacteria are almost exclusively seen as single bacteria in infected macrophages whereas wild-type *M*. *marinum* was often seen in clusters (Fig 4D, S7C Fig, S1 & S2 Movies), which is in line with our flow cytometry results. One potential confounding factor in our experiments was the trend of increased ubiquitin co-localization with larger cluster sizes for wild-type *M*. *marinum* (S7A Fig). To exclude the possibility that our results of ubiquitin recruitment are biased because of bacterial clustering, we also assessed the mean fluorescent intensity of FK2 co-localization of cells infected with one bacterium (S7A & S7B Fig). THP-1 macrophages infected by a single *M*. *marinum* bacterium showed the same trends of ubiquitin association as observed in the whole population (S7B Fig), suggesting that the observed phenotypes are caused by differences in capsule integrity and not differences in bacterial cluster size (S7C Fig). Together, these data show a clear effect on the virulence of *M*. *marinum* in the early stages of infection through reduced ubiquitin co-localization, which suggests a defect in phagosomal rupture. To examine whether the *ppe10* mutant was able to escape the phagosome at later timepoints, we performed electron microscopic analysis. THP-1 cells infected with either WT bacteria or the *ppe10* mutant were fixed 48 hours post infection, sectioned and analyzed by cryo-immunogold electron microscopy to determine the subcellular localization of the bacteria. This analysis confirmed that both the WT and the mutant were able to translocate efficiently from phagolysosomes into the cytosol at this later timepoint, as nearly half of the bacteria were detected in the cytosol (S8 Fig). These data show that, although there seems to be a delay in the association with ubiquitin early in the infection of host macrophages, the *ppe10* mutant is able to escape the phagosome at later timepoints, similar to what has been shown previously for the ESX-5 mutant 7C1 (*espG*_*5*_::*tn*) [20].

### Zebrafish embryo infection experiments

**Fig 5:**
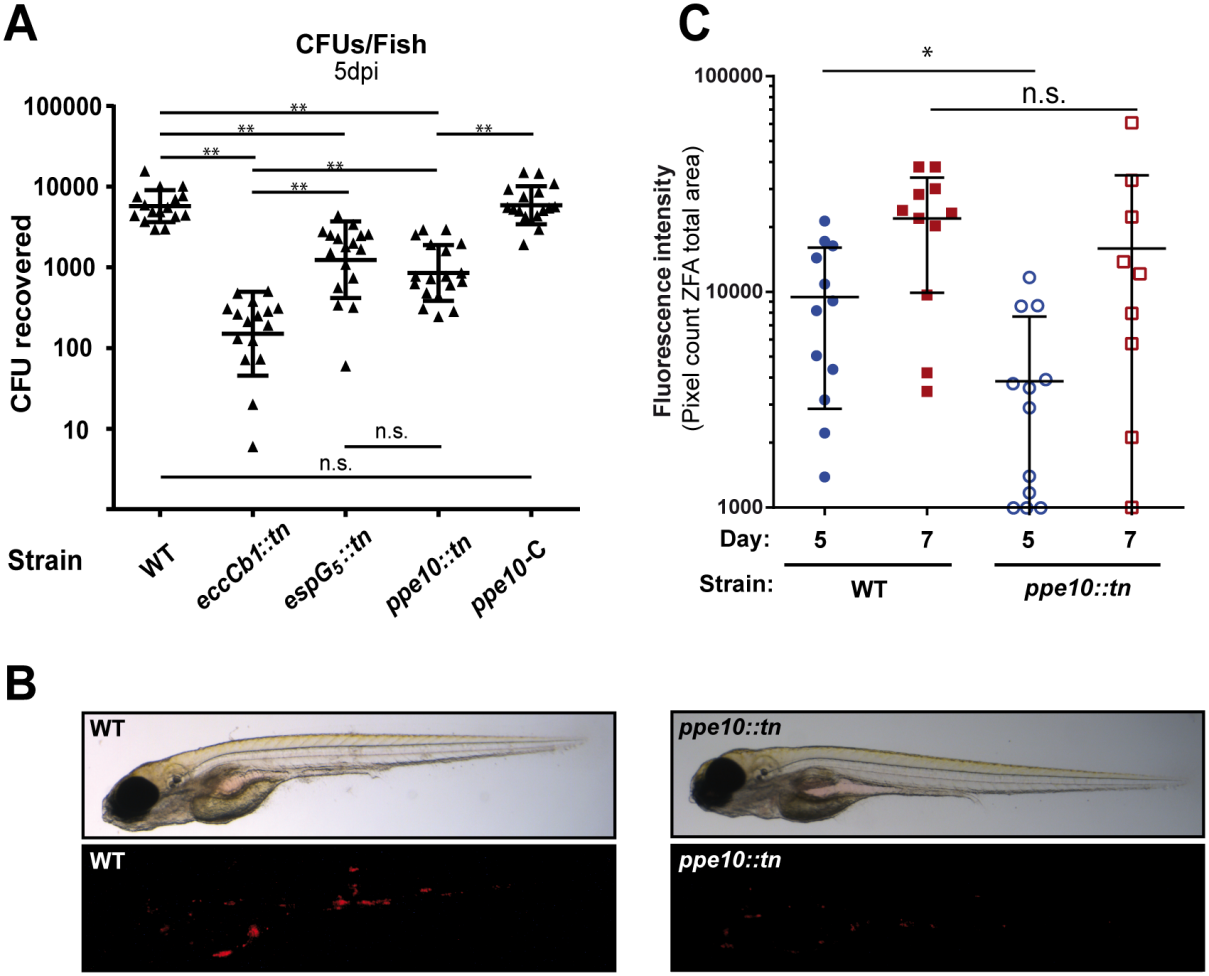
The *M*. *marinum ppe10*::tn mutant is attenuated in zebrafish embryo infections. A) *M*. *marinum* E11 wild type strain and the isogenic mutants *espG*_*5*_::*tn*, *eccCb*_*1*_::*tn* and *ppe10*::*tn*, as well as the complemented *ppe10*::*tn* strain (PPE10-C), were pre-cultured in liquid medium containing Tween-80. 50–100 CFUs of bacteria were injected in the bloodstream of zebrafish embryos at 28 hours post fertilization. Embryos were homogenized five days post infection and plated to establish the number of CFU per embryo. Three independent experiments of six embryos per group were performed and the data were pooled. B) Visualization of *M*. *marinum* infection of zebrafish embryos. Wild-type *M*. *marinum* or *ppe10*::*tn* containing the plasmid pSMT3::*mCherry* was injected in the bloodstream of zebrafish embryos as described above. After 5 days of infection the embryos were examined by brightfield (top) or fluorescence (bottom) microscopy. C) Quantification of fluorescence in infected zebrafish embryos. *M*. *marinum* wild type (closed symbols) and the *ppe10*::*tn* (open symbols) mutants expressing *mCherry* were injected in the bloodstream of zebrafish embryos 28 hours post fertilization. Images were acquired by fluorescence microscopy at day 5 (blue) and 7 (red) post infection and were analyzed for fluorescent intensity by dedicated software [43]. Differences on day 5 and day 7 were analyzed by GraphPad Prism software using Mann-Whitney two-tailed test. * = *p*<0.05, ** = *p*<0.01, n.s. = not significant.

A previous report described the temporary attenuation of the *espG*_*5*_::*tn* mutant in zebrafish embryos [42]. We set out to test whether this phenotype of delayed infection was due to reduced capsule integrity and defective secretion of PPE10. Zebrafish embryos were infected with wild-type *M*. *marinum*, *espG*_*5*_::*tn*, *eccCb*_*1*_::*tn*, *ppe10*::*tn* and *ppe10*::*tn*-C and 5 days after infection embryos were homogenized and colony forming units (CFUs) were quantified (Fig 5A). As expected, the ESX-1 mutant strain *eccCb*_*1*_::*tn* showed strong attenuation, with an approximate 100-fold reduction in recovered CFUs after five days. As previously described, the *espG*_*5*_::*tn* strain exhibited a significant reduction in recovered CFUs (log transformed mean difference compared to wild-type 0.6649, *p*<0.001). The *ppe10*::*tn* mutant was attenuated to a similar extent as *espG*_*5*_::*tn* (log transformed mean difference compared to wild-type 0.8275, *p*<0.001). The difference between *espG*_*5*_::*tn* and *ppe10*::*tn* was non-significant, while the complemented *ppe10*::*tn* strain was comparable to wild-type *M*. *marinum*. Although both the *espG*_*5*_ and *ppe10* mutants were attenuated, they showed significantly (*p*<0.001) higher CFU counts as compared to the ESX-1 mutant, indicating that strains with impaired capsular integrity are partly, but not completely attenuated. These differences were quantified by analyzing images of infected zebrafish (Fig 5B) by dedicated software [43]. At 5 days post infection, a significant difference in infection could be measured (Fig 5C), confirming the CFU quantification of infected fishes. To test whether this attenuation was specific for the early stages of infection as was described before [42], we also quantified the infection in the same fish embryos at 7 days post injection. At this timepoint differences in infection were no longer significant (Fig 5C). Together these data indicate that the earlier reported virulence defects of the ESX-5 deficient strain in initial stages of infection [20,42] could largely be attributable to the loss of PPE10 secretion and is therefore probably caused by reduced capsule integrity of these mutants.

## Discussion

In this study, we demonstrate that the ESX-5 secretion system plays a major role in capsular integrity of pathogenic mycobacteria and that the ESX-5 substrate PPE10 seems to be a key player in this phenomenon. The existence of the mycobacterial capsule has already been postulated a long time ago, but remained controversial [7,44] until the recent visualization of intact, plunge-frozen mycobacteria by cryo-EM [6]. These experiments showed that detection of the capsule was only possible when the bacteria were grown without detergent, indicating that this capsule is a labile structure, prone to disruption [6]. Moreover, different components of the mycobacterial capsule have different susceptibilities to the presence of detergent [6, this study] or for extraction by mechanical methods [45]. For instance, whereas EspE, the major capsular protein component of *M*. *marinum*, is only present on the cell surface in high amounts when cells are grown without Tween-80, PE_PGRS proteins can be identified in the capsule-enriched fraction in both conditions. These data correspond with earlier reports [6,45] suggesting that the mycobacterial capsule might consist of several layers with different susceptibilities for disruption. In addition, there is also a clear difference in capsule stability and composition between different mycobacterial species [6]. Here we show that most capsular components of *M*. *marinum* that were examined were more susceptible to disruption by detergents in strains defective in ESX-5 secretion or in the ESX-5 substrate PPE10. This is confirmed by cryo-EM analysis, which showed that the *esx-5* mutant has a ruffled morphology of the capsule. This suggests that ESX-5 substrate(s) are not involved in capsule biogenesis, but play a direct or indirect role in the anchoring of the capsule. Both the *ppe10*::*tn* and *espG*_*5*_::*tn* mutants also possessed a significantly less hydrophobic cell-surface. This reduced hydrophobicity could not be attributed to altered levels of any of the analyzed (glyco-)lipids, suggesting that either a protein or carbohydrate moiety is responsible for this phenotype or that these mutants have a defect in lipid transport. Reduced cell-surface hydrophobicity could very well explain the reduced binding of the relatively hydrophobic PE_PGRS proteins.

Our data show that esx-5- or ppe10-mutations in *M*. *marinum* result in capsular defects, reduced hemolytic activity when grown with tween, a delay in ubiquitin association during host cell infection and attenuation in zebrafish embryos. Our data provide an explanation for the previously observed delay in phagosomal escape of the *esx5* mutant [20]. However, it should be noted, that this delay in phagosomal rupture might not apply to *M*. *tuberculosis*, since this species already shows a delayed escape as compared to *M*. *marinum* (*i*.*e*. significant escape after 48 hours instead of 2–4 hours) [20]. In contrast to our cell-infection experiments, the preparation of zebrafish embryo injection stocks requires bacteria to be brought into single-cell suspension by a wash step with 0.5% Tween-80. It can therefore be assumed that all mutants lack an intact capsule at the moment of infection, as was shown for wild-type bacteria by Sani et al. [6], and that differences in infection are therefore probably related to the *in vivo* synthesized capsule. Throughout these experiments, the *ppe10* mutant showed more pronounced phenotypes than the ESX-5 mutant. A possible explanation for this could be that some residual secretion of native PPE10 might occur in the ESX-5 mutant that was used (*espG*_*5*_::*tn*), as this mutant is strongly reduced but not completely devoid of secretion [24,46]. Please note that a completely negative ESX-5 secretion mutant is lethal, as we and others have recently reported for different mycobacterial strains/species [25,47].

The ESX-1-dependent membrane disruption and phagosomal escape of pathogenic mycobacteria has been extensively studied in recent years [13–15,22,48]. The importance of the ESX-1 system in this process could be confirmed using imaging flow cytometry, which measured the co-localization of ubiquitin with intracellular *M*. *marinum*. This new procedure allows a rapid quantitative approach to study the subcellular localization and co-localization of host factors with mycobacteria inside the host cell. Interestingly, the co-localization of *M*. *marinum* with ubiquitin correlated very well with surface exposed EspE, as detected by our various extraction and surface labeling methods. Although EspE is the most abundant capsular protein in *M*. *marinum*, it is not known whether this ESX-1 substrate is responsible for the observed phenotypes in *in vivo* experiments. Other studies have implicated EsxA as the membrane disrupting factor of the ESX-1 secretion system [49,50]. In our experiments, surface localization of EsxA did not correlate with ubiquitin co-localization and hemolysis of the strains, indicating that possibly other ESX-1 secreted substrates are involved in this process.

Our data shows that capsular defects are linked with the attenuation of *M*. *marinum*, probably through the differentially localized ESX-1 substrates. However, possibly also other effects on virulence could be present. For instance, the capsular polysaccharide α-glucan [51] and the glycolipid ManLAM [52] were previously shown to interact with pathogen recognition receptors and have immune-modulating properties [51,52]. Furthermore, *M*. *bovis* BCG and *M*. *tuberculosis* grown without detergent and therefore possessing an intact capsule were shown to induce higher levels of protective immune responses in a mouse model [9]. One study identified a *ppe10* transposon mutant of *M*. *bovis* BCG in a screen for mutants that were defective in avoiding phagosome acidification [53]. This phenotype led to an attenuation of BCG-*ppe10*::*tn* compared to wild-type BCG after 4 and 6 days of infection. Since BCG does not possess a functional ESX-1 secretion system, these results show that this defect in the arrest of phagosome acidification is not coupled to ESX-1 substrates. Another interesting observation is the effect we observed of capsule integrity on cell clumping. Both the *espG*_*5*_::*tn* and *ppe10*::*tn* mutant showed a single cell phenotype, indicating that the capsule is an important component in cell clumping. This phenotype could be explained by the reduced surface hydrophobicity of these mutants. Mycobacterial clumping has also been associated with trehalose dimycolate (cord factor) [Reviewed in 54] and a defect in lipooligosaccharide (LOS) biosynthesis [55]. However, our lipid analyses show that the biogenesis of these moieties is not affected in the *esx5* or *ppe10* mutant strains. It is possible however, that the ESX-5 system and/or PPE10 play a role in the export of these extracellular (glyco)lipids, leading to the observed phenotypes on capsular morphology and cell aggregation.

What is the molecular mechanism for the role of PPE10 in capsular integrity? The reduced surface hydrophobicity seems important in explaining the phenotypes of PPE10 deficient strains. As discussed above, PPE10 could have a role in the export of a lipid or carbohydrate moiety important for the integrity of the capsule. Alternatively, PPE10 could be a structural component of the capsule by binding both the cell-surface and other capsular components. PPE10 is detected on the cell-surface of *M*. *marinum* in relatively high amounts by mass-spectrometry [25], even though sequence coverage of the protein is relatively poor due to the paucity of trypsin cleavage sites. This indicates that the protein is probably present in high amounts on the bacteria. PPE10 is also expressed *in vivo*, as it is detected in the lungs of infected guinea pigs at both 30 and 90 days post infection [56], and expression levels seem to be relatively constant at different time points in a mouse infection model [57]. There are close orthologues of *ppe10* present in other pathogenic mycobacteria such as *M*. *tuberculosis*, *M*. *ulcerans*, and *M*. *bovis* [26], which likely have the same function in these species. PPE10 orthologues of pathogenic mycobacteria contain pentapeptide repeat domains (pfam01469 [58]) and are therefore grouped in the PPE subfamily of major polymorphic tandem repeat (MPTR) proteins [59,60], of which PPE10 is thought to be the most ancient [26]. Unfortunately, no role for PPE-MPTR proteins has been described. The strong phenotype of the *ppe10*::*tn* strain suggests that there is no redundancy in the function of PPE10 and other PPE-MPTR proteins of *M*. *marinum*. Therefore, it is possible that the MPTR domains are only structural domains not directly involved in protein function. It is intriguing that PPE10, which is only present in slow-growing pathogenic mycobacteria, is so important for capsular integrity, since fast-growing mycobacteria also contain a capsule [6]. However, other pentapeptide repeat containing proteins of fast-growing mycobacteria could possibly perform a similar function. Intriguingly, the MYCSM_05776 protein of *M*. *smegmatis* strain JS623 contains a penta-peptide repeat with 40–50% identity compared to PPE10. However, this protein contains a classical signal sequence instead of a PPE domain. Such a dichotomy between PE/PPE extensions in slow-growing mycobacteria and classical secretion signal extensions in fast-growing mycobacteria is also observed for the surface lipase LipY [30].

Finally, our data illustrate that the different phenotypes attributed to the different T7S systems can be interdependent. We have shown *esx-5* and *ppe10* mutations of *M*. *marinum* result in reduced amounts of surface localized ESX-1 substrates, defects in erythrocyte membrane rupture and possibly in delayed phagosomal rupture. Because ESX-1 has been shown previously to be involved in phagosomal escape [14,15], these phenomena are probably linked. These data help to elucidate previously-described but unexplained results [24,42,53]. These new results open the way to study the role of the mycobacterial capsule in virulence by new approaches and might uncover multiple other effects on virulence that are suggested by literature [51–53]. The surface localization of PPE10, combined with the multiple possible effects on mycobacterial pathogenesis of this protein, make this an interesting candidate for further analysis to unravel virulence mechanisms of mycobacterial pathogens.

## Materials and Methods

### Strains and growth conditions

All *M*. *marinum* strains that were used were derived from the wild-type strain E11 [61,62], except for the quality control of the anti-EspE antibody for which M^USA^ and its ΔRD1 mutant were used. The ESX-5 secretion mutant *espG*_*5*_::*tn* is previously described as transposon mutant 7C1 and has a transposon insertion in *mmar_2676* (*espG*_*5*_) [24]. The mutant *eccCb*_*1*_::*tn* was identified previously as an ESX-1 mutant [43]. Strain CDC1551 and the isogenic transposon mutant in *eccC*_*5*_ (JHU1783-2086) were described previously [35] and were kindly provided by BEI-resources [63]. All strains were cultured on Middlebrook 7H10 plates or in Middlebrook 7H9 medium (Difco) containing ADC supplement and, when required, 0.05% Tween-80 and the appropriate antibiotic selection markers (25 μg/ml kanamycin (Sigma) and/or 50 μg/ml hygromycin (Roche)). *M*. *marinum* was incubated at 30°C whereas *M*. *tuberculosis* strains were cultured at 37°C.

### Transposon mutagenesis

Transposon mutagenesis and secretion analysis by double filter assay was performed as described by van der Woude et al. [55]. Briefly, *M*. *marinum* strain E11 was infected with the mycobacteriophageφMycomarT7, containing the mariner-like transposon Himar1 [64]. The transposon library was plated on 7H10 plates covered by a nitrocellulose filter. After colonies were formed, a second filter was placed underneath and was incubated overnight. The second filter was stained by an anti-PE_PGRS antibody (7C4.1F7) [24] and colonies were screened for increased secretion. Positive colonies were rescreened on a second double-filter assay, before the localization of the transposon insertion was determined using ligation-mediated PCR [36,65].

### Construction of plasmids

The *mmar_0761* (*ppe10*) gene was amplified from *M*. *marinum* E11 genomic DNA using primers LA_0761-fw (TTTGCTAGCGTGCAAACCCGCATT) containing a NheI restriction site and LA_0761-rv (AAAGATATCAGCATAATCAGGAACATCATACGGATACTAC TCCGTGCGCAGCGGCA) containing an EcoRV restriction site and a stop codon before the HA sequence. For the construct with an HA-tag, reverse primer LA_0761-HA-Rv (TGCCGCTGCGCACGGAGTATCCGTATGATGTTCCTGATTATGCTTAGGATATC) was used. PCR products were cloned into the pSMT3 vector [30] by replacing *lipYtub* with the indicated constructs after restriction with EcoRV and NheI. All constructs were checked by sequence analysis.

### Immunoblot and secretion analysis

Bacteria were grown until mid-logarithmic phase and were washed and cultured overnight in 7H9 medium containing glycerol and dextrose, but without bovine serum albumin (BSA) (Sigma). Depending on the experimental conditions, 0.05% Tween-80 was added to the cultures. After overnight culture, supernatants were filtered using a 0.02 μm filter, concentrated by TCA precipitation and washed with acetone. Bacteria were harvested and washed once with PBS. Aliquots were taken for whole cell lysates and for Genapol X-080 extraction of capsular proteins. Genapol extraction was performed by incubating bacteria 30 minutes in 0.05% Genapol X-080 in PBS under head-over-head rotation at room temperature. Genapol X-080 extractable fractions were diluted in 5X concentrated solubilization/denaturation (S/D) buffer. Whole cell lysates and Genapol X-080 extracted cell residues were solubilized in S/D buffer and sonicated to lyse the cells. All samples were boiled for 10 minutes at 95°C before loading on SDS-PAGE. After SDS-PAGE, proteins were transferred to nitrocellulose membranes by western blot, which were subsequently incubated with different antibodies followed by electrochemiluminescence. Primary antibodies used were: anti-PE_PGRS antibody (7C4.1F7) [24]; anti-GroEL2 (CS44, Colorado state university); anti-EsxA (Hyb76-8) [66]; anti-influenza hemagglutinin (HA) epitope (HA.11, Covance) and polyclonal anti-EspE (This study, produced as described in [67]). Secondary goat-anti-mouse (American Qualex) or goat-anti-rabbit (Rockland) horseradish peroxidase labeled antibodies were used and visualized by enhanced chemiluminescence (ECL prime, Amersham).

### Bacterial flow cytometry analysis and surface labeling

*M*. *marinum* strains expressing pSMT3::*mCherry* [39], were pre-cultured in 7H9 medium with Tween-80 and incubated overnight in the presence or absence of Tween-80. Cells were harvested by centrifugation and were incubated with an anti-EspE polyclonal antibody [67], followed by a FITC-labeled secondary goat anti-rabbit antibody (BD bioscience). Bacterial cells were acquired on a BD AccuriC6 flow cytometer (BD biosciences). Particle size estimation was performed by comparing it to beads from the flow cytometry size calibration kit (Life Technologies). 20.000 red fluorescent (610/20 nm) events were acquired in a gate on side scatter and forward scatter that corresponded to particle sizes of single bacteria. Green fluorescence (530/30 nm) of the gated cells was quantified and plotted.

### Electron microscopy

For immunogold electron microscopy, *M*. *marinum* strains were inoculated from an exponentially growing culture to 7H9 medium with or without detergent at 0.35 OD_600_/ml and incubated without agitation. After overnight incubation, 5 OD-units of bacteria were collected by centrifugation and fixed for 24 hours in 0.2M PHEM (final concentrations: 120mM PIPES, 50mM HEPES, 4mM MgCl_2_ 20mM EGTA) buffer with 4% paraformaldehyde and 0.4% glutaraldehyde. *M*. *tuberculosis* was grown until mid-logarithmic phase in the presence of 0.05% Tween-80. At this point, samples were collected and fixed as described above, while cells were also re-inoculated in a medium lacking Tween-80 and incubated for 6 days after which samples were collected and fixed. Fixed bacteria were incubated on carbon coated formvar grids for 5 minutes. Grids were stained with different antibodies: anti-EspE polyclonal rabbit serum (produced as described in [67], quality control in S3A Fig); anti-PIM_6_ (F183-24) [6,33]; anti-ManLAM (55.92.1A1) [6,11]. Gold-labelled secondary antibodies (Utrecht University) were used and surface labelling was visualized on a Tecnai 12 electron microscope (FEI, Eindhoven, the Netherlands). Plunge freezing and Cryo-EM was performed as described in Sani *et al*. 2010 [6].

### Cell-surface hydrophilicity

Cell-surface hydrophilicity was measured as previously described [36,68]. Briefly, *M*. *marinum* and the isogenic transposon mutants, as well as the *ppe10*::*tn* complemented strain, were grown in 7H9 medium supplemented with Tween-80 after inoculation from plate. Bacterial suspensions of 1.0 OD_600_ in 1ml of PBS were incubated 2 hours with or without 0.5% (v/v) Xylene. The organic phase was taken from the aqueous phase and was discarded. The aqueous phase was measured for optical density (OD_600_) and compared to samples without Xylene. Without Xylene, the OD of the aqueous phase never differed more than 0.1 OD from the starting inoculum. Data ware gathered from three independent experiments, which were all performed in triplicate and were compared via statistical analysis using the GraphPad Prism software (non-parametric test for multiple comparisons against a single control).

### Mycobacterial lipids extraction and TLC analysis

*M*. *marinum*, *espG*_*5*_::*tn*, *ppe10*::*tn* and the *ppe10*::*tn*-complemented strains were cultured in liquid 7H9 as describe above and 50 OD units biomass was collected. The extraction of the cell envelope lipids was performed in three steps to harvest the apolar, polar and mycolic acid lipid fractions as previously described [69,70]. The different lipid fractions were analyzed with 1D-TLC or 2D-TLC as earlier described [71]. Briefly, equal amounts of the lipids were spotted on silica-60 (Merck) TLC plates and lipids were separated by 1D or 2D-TLC on diverse solvent systems (see below). Subsequently, the lipids were visualized by using 5% molybdophosphoric acid (MPA) in ethanol or orginol in 20% H_2_SO_4_ coloring agents and TLC-plate charring at 150°C for 10 minutes. The apolar lipids were separated by 1D-TLC, heptane/di-isopropyl ether/ acetic acid (60:40:3, v/v/v) and chloroform/methanol (90:10, v/v). 2D-TLC, solvent system A; petroleum ether/ethyl acetate (98:2 v/v) and petroleum ether/ acetone (98:2 v/v). The polar lipids were separated on 1D-TLC, chloroform/acetic acid/methanol/water (40:25:3:6, v/v/v/v) and 2D-TLC system E; chloroform/methanol/water (20:10:2, v/v/v) and chloroform/acetic acid/methanol/water (40:25:3:6, v/v/v/v). The mycolates were analyzed with 1D-TLC hexane/ethyl acetate (19:1, v/v).

### Hemolysis

Hemolysis experiments were performed mostly as described by Smith *et al*. [17]. Shortly, *M*. *marinum* strains were grown in a liquid pre-culture in 7H9 medium supplemented with 0.05% Tween-80 until mid-logarithmic phase was reached. All strains were washed once in 7H9 medium without Tween-80 and inoculated in 7H9 with- or without Tween-80 at 0.35 OD_600_/ml. After overnight culture, bacteria were collected by centrifugation, diluted in PBS, and quantified by absorbance measurement at OD_600_. It was previously estimated that 1 ml of 1.0 OD_600_ of bacteria contain 2 * 10^8^ bacteria. In parallel, defibrinated sheep erythrocytes (Oxoid—Thermo Fisher, the Netherlands) were washed 5 times and diluted in fresh DMEM medium without phenol red (Gibco, Life technologies). Erythrocytes were counted using a Bürker-Türk hemocytometer and 8.3*10^8^/ml erythrocytes and 2.08*10^9^/ml bacteria were added in a round-bottom 96 well-plate and gently centrifuged for 7 min. at 600 RPM in a swing out-plate centrifuge and incubated at 30°C for 3 hours. After incubation, cells were brought into suspension and centrifuged. Supernatant was transferred to a flat-bottom 96-wells plate and absorbance at 405nm was measured to quantify hemoglobin release.

### Cell infections and ubiquitin co-localization

*M*. *marinum* E11 and transposon mutants *eccCb*_*1*_::tn, *espG*_*5*_::*tn* and *ppe10*::*tn* strains expressing pSMT3::*mCherry*[39], were cultured overnight with or without 0.05% Tween-80 as described above. THP-1 cells were maintained in RPMI 1640 GlutaMAX supplemented with 10% FCS at 37°C and 5% CO_2_. Monocyte differentiation was started 5 days prior to infection by incubation with 10ng/ml PMA for 48 hours. For infection studies, THP-1 cells were seeded in 6 well plates at a density of 2*10^6^ cells per well. After differentiation cells were infected at an MOI of 10 for 2 hours. Subsequently, cells were washed 3 times with warm PBS and incubated for an additional hour in complete RPMI media. To analyze ubiquitin recruitment to the bacteria, cells were trypsinized and fixed in 2% PFA for 15 minutes. All staining procedures of the fixed THP-1 cells occurred in the presence of 0.1% Saponin. Cells were blocked for 45 minutes with 1% BSA and stained for ubiquitin with the FK2 antibody (Millipore, 1:200 dilution) in the presence of 1% BSA for 1 hour. Cells were subsequently washed twice and labeled with a secondary antibody Goat anti Mouse Alexa488 (Life technologies, 1:200 dilution) for 30 minutes followed by 2 washes with PBS. Cells were post-fixed with 2% PFA before analysis.

### Imaging flow cytometry

Cells were analyzed on the ImageStreamX100 (Amnis-Merck Millipore) imaging flow cytometer as previously described [72,73]. A minimum of 15,000 cells were acquired per sample. For standard acquisition, the 488 nm laser line (for FK2) was set at 100 mW and the 561 nm laser line (for mCherry) was set at 200mW. Single staining controls were used to generate compensation files that allowed correction of spectral overlap. Co-localization scores were calculated as previously described [72] using the *bright detail similarity R3* feature in the Ideas software. This feature corresponds to the logarithmic transformation of Pearson’s correlation coefficient of the localized bright spots with a radius of 3 pixels or less within the whole cell area in the two input images. Bacterial counts were calculated using the peak mask in combination with the spot count feature as previously described [73].

### STED super-resolution microscopy

Stimulated Emission Depletion microscopy (STED) was performed on a Leica TCS SP8 STED 3X microscope, Leica Microsystems, (Wetzlar, Germany). Samples were irradiated with a pulsed white light laser at wavelengths 488 nm and 587 nm with a power of 20%, which translates to 84mW. A continuous wave STED laser line at a wavelength of 660nm and 210mW power (20%), Leica Microsystems, was used for depletion of the 488nm fluorophore, reaching a lateral resolution of ~70nm. The signal was detected using a gated Hybrid Detector (HyD), Leica Microsystems, with a gain of 120% and a range of 503–569 nm for the green channel. The red channel was set at a gain of 142% and a range of 603–651 nm. STED images were acquired using a dedicated oil objective with 100x magnification and a numerical aperture of 1.4, Leica Microsystems. A Z-stack was made with a step size of 200nm and pixel size of 24nm x 24nm, optimized using Nyquist Calculator (SVI Scientific Volume Imaging, Hilversum, the Netherlands). Finally, deconvolution was performed with Huygens Professional Software (SVI).

### Infection of THP1 cells and immuno-gold labeling for electron microscopy

THP-1 cells were seeded in 7.5 cm diameter flasks and infected with *M*. *marinum* E11 or *ppe10* mutant similar to already described in Abdallah et al 2011 [20]. In short, cells were incubated with bacterial culture (OD_600_ 0.5) for 1h, then supernatant was removed to remove extracellular bacteria and infected cells were washed three times with medium. Subsequently, THP1 cells were incubated in fresh medium with FCS at 33°C and 5% CO_2_ for 24 or 48 hours. Cells were then fixed with paraformaldehyde and gluteraldehyde for 2 hours. After processing for cryo-sectioning, 60–70 nm thick sections were cut at -120°C, and sections were immuno-labeled with CD63 (M1544 Sanquin) and protein-A 10 nm gold (EM laboratory, Utrecht University) and stained with uranyl acetate. Analysis of the sections was performed using a FEI Tecnai 12 transmission electron microscope at 100kV.

### Zebrafish experiments

Zebrafish (*Danio rerio*) embryo experiments were performed as described previously [43]. Briefly, *M*. *marinum* strains were grown to an OD_600_ of 1.0, washed in PBS and declumped in 0.5% Tween-80. The amount of colony-forming units (CFU) was verified by plating on 7H10 plates. Zebrafish embryos were injected 28 hours post fertilization, with 100–200 CFUs per embryo. Five days post infection, embryos were sedated after which the embryos were homogenized in 100μl of 5% SDS solution in PBS. Homogenized embryos were treated with Mycoprep (BBL), to decontaminate the sample and different concentrations were plated on 7H10 plates without antibiotic additives. 10 days after plating, colony-forming units were counted. The experiment was performed on three individual time-points with six embryos per group per experiment. To visualize the infection, zebrafish embryos were injected with strains *M*. *marinum* strains expressing *mCherry* from the *pSMT3*::*mCherry* plasmid as described above. Images were acquired on days 5 and 7 after infection on a fluorescence microscope (Leica MZ16FA) with a specialized camera (Leica DFC420C) and analyzed by dedicated software as previously described [43].

### Ethics statement

All procedures involving embryonic zebrafish (*Danio rerio*) were performed in compliance with local animal welfare laws. All zebrafish were maintained according to standard protocols (zfin.org) and breeding of adult dish was authorized by the Animal Experimental licensing Committee (DEC) of the VU University Medical centre. All experiments followed the international guidelines specified by the EU animal protection directive 86/609/EEC, which allows use of embryonic zebrafish until a free-living stage (approximately 5–7 days after fertilization) and were therefore approved by the DEC of the VU University Medical centre (Amsterdam, the Netherlands).

## Supporting Information

S1 Fig Complementation of *ppe10*::*tn*. Immunoblot analysis of *ppe10*::*tn* complementation by overexpression of *mmar_0761* on the episomal plasmid pSMT3 under the control of the *hsp60* promoter. P = Whole cell lysate, GP = Genapol X-080 treated cells, GS = Supernatant of Genapol X-080 treated cells, CF = Culture filtrate. Complementation was performed with or without a C-terminal HA-tag fused to *mmar_0761*. The PE_PGRS supersecretor phenotype of *ppe10*::*tn* was restored upon introduction of native- or HA-tagged *mmar_0761*. However, surface localization of EspE was not restored with HA-tagged *mmar_0761*-HA, but was partially restored by *mmar_0761*.
(TIF)

S2 Fig Mutations affecting capsular integrity influence cellular aggregation of *M*. *marinum*. A) Flow cytometry plots showing forward scatter (FSC—x-axis) and side scatter (SSC—y-axis) of indicated *M*. *marinum* mutants in the presence or absence of 0.05% Tween-80. Gate P2 was set as a measurement of mycobacterial ‘clumping’, with the percentage of gated cells depicted in red. The presence of Tween-80 reduces clumping in all mutants. However, *espG*_*5*_::*tn* and *ppe10*::*tn* show considerably less clumping compared to the wild-type, while the *eccCb*_*1*_::*tn* shows larger aggregates. B) Size estimation of analyzed particles. Black arrows indicate sizes of calibration beads used as a reference for particle sizes. C) Quantification of gated cells of plots depicted in [A].
(TIF)

S3 Fig Electron microscopy surface labeling of capsular components. A) Quality and specificity control of the anti-EspE polyclonal antibody. *M*. *marinum* M^USA^ or the RD1 deletion strain Δ*mmar_3871–9* [32] (ΔRD1) were labeled by the anti-EspE serum or by pre-immune rabbit serum followed by a gold-labeled secondary antibody. Specific surface labeling was only observed in the wild-type bacteria labeled with the anti-EspE serum, indicating that this antibody specifically labels EspE. B) Quantification of EspE surface labeling of *M*. *marinum* strains. EspE surface labeling could be detected in wild-type *M*. *marinum* irrespective of the presence of Tween-80. EspE surface labeling was reduced to levels of the negative control *eccCb*_*1*_::*tn* when *espG*_*5*_::*tn* was grown with Tween-80. C) Quantification of electron microscopy surface labeling of *M*. *tuberculosis* wild-type strain CDC1551 or an isogenic ESX-5 mutant strain *eccC*_*5*_::*tn* by an anti-Mannose-capped-lipoarabinomannan (ManLAM) antibody. Surface labeling of ManLAM is reduced in CDC1551 in the presence of Tween-80 and is markedly less in the *eccC*_*5*_::*tn* strain irrespective of the presence of Tween-80. D) Transmission electron microscopy images of representative images from the dataset depicted in C. The length of the black scale bars represents 500 nm.
(TIF)

S4 Fig 1D-TLC analysis of *ppe10* and esx5 mutants reveals no differences in (Glyco-) lipid levels. A) Apolar lipids fractions were separated by TLC using heptane/di-isopropyl ether/acetic acid (60:40:3, v/v/v) solvent and analyzed for the acyl-glycerol classes. The arrows indicate mono- (MAG), di- (DAG) and tri- (TAG) acyl-glycerols respectively. B) Mycolic acid fractions were separated by TLC using hexane/ethyl acetate (19:1, v/v) solvent. Arrows indicate the α-, methoxy- and keto- forms of the mycolic acids respectively, as well as the fatty acid methyl esters (FAMEs). C) Polar lipids separated by 1D-TLC using chloroform/acetic acid/methanol/water (40:25:3:6, v/v/v/v) solvent. Phosphatidylinositol mannosides (PIM) containing 2 (PIM_2_) and 6 (PIM_6_) mannose residues are respectively depicted. D) 1D-TLC of apolar lipids separated by chloroform/methanol (90:10, v/v). Arrows indicate phenolic glycolipids (PGL) and trehalose dimycolate (TDM). The lipids were visualized by 5% MPA in ethanol (A and B) or 5% orginol in 20% H_2_SO_4_ (C and D) and subsequent plate charring. PPE10-C = *ppe10*::*tn* expressing pSMT3::*mmar_0761*.
(TIF)

S5 Fig 2D-TLC analysis of *ppe10* and esx5 mutants reveals no differences in (Glyco-)lipid levels. A) Analysis of PDIM lipids. Apolar lipids were separated by 2D-TLC with petroleum ether/ethyl acetate (98:2 v/v) and petroleum ether/ acetone (98:2 v/v) solvents respectively and were visualized by spraying with 5% MPA and plate charring. Location of PDIMs and TAGs are indicated by black arrows. B) 2D-TLC analysis of LOS and PIM glycolipids. Polar lipids were separated by TLC using chloroform/methanol/water (20:10:2, v/v/v) and chloroform/acetic acid/methanol/water (40:25:3:6, v/v/v/v) solvents respectively and were visualized by orginol spraying and plate charring. PIMs and different LOS fractions are indicated with the black line and arrows respectively. *ppe10-C* = *ppe10*::*tn* expressing pSMT3::*mmar_0761*.
(TIF)

S6 Fig Analysis of co-localization of ubiquitin with bacteria by imaging flow cytometry. A-D) THP-1 macrophages infected with the indicated strain of *M*. *marinum* expressing *mCherry* were stained with the FK2 antibody recognizing poly-ubiquitin and were analyzed by imaging flow cytometry. Bacteria were pre-cultured in the presence (Red lines) or absence (Blue lines) of Tween-80. Relative co-localization of green and red fluorescence was quantified per particle (X-axis). Cells within gate R1 (green line) were seen as positive for co-localization of ubiquitin and bacteria for further analyses. Data of two independent experiments were pooled and analyzed together. E) The fluorescence intensity data depicted in the histogram plots (S6A–S6D Fig) was fitted to a one phase decay (Y = (Y0—Plateau)*exp(-K*X) + Plateau) with the constrain of the plateau set to 0. The goodness of fit for all data sets was greater than 0.9. The rate constant (K) was plotted and 95% CI intervals are shown. Non-overlapping confidence intervals are necessarily significantly different. The significantly higher rate constant for wild-type (Blue) and *espG*_*5*_::*tn* (Green) in the presence of Tween, indicate fewer ubiquitin associated bacteria. The highest K value (lowest amount of ubiquitin associated bacteria) was observed for the *ppe10*::*tn* mutant (Black), which was independent for the presence (Filled bars) or absence (Striped bars) of Tween.
(TIF)

S7 Fig Co-localization of ubiquitin and bacteria is correlated with bacterial cluster size, but also by differences in capsular ESX-1 substrates. THP-1 macrophages were infected by the indicated strains of *M*. *marinum* expressing mCherry and were pre-cultured in the presence or absence of Tween-80. Infected cells were stained with the FK2 antibody and a FITC-labeled secondary antibody. A) Cells were analyzed by imaging flow cytometry and were sorted for the amount of bacteria per cell (Y-axis) and the intensity of FK2 staining (X-axis). Color coding indicates the density of analyzed particles with indicated fluorescent intensities. The blue rectangle indicates the gate used to analyze macrophages infected by a single bacterium. B) Analysis of the mean fluorescent intensity (MFI) of FK-2 staining measured by the FITC signal (Y-axis) of gated cells that contain only a single bacterium. Relative MFI data correlated very well with data obtained in other assays and analyses (Fig 4A and 4B) indicating that the number of bacteria per macrophage is not the driving force behind the observed phenotype of ubiquitin co-localization. C) Average number of bacteria per infected macrophage. SEM values are shown from three independent experiments. The Kolmogorov-Smirnov test (data is not Gaussian distributed) was used to determine statistical significance between identical strains and between wild type and mutant strain grown in the presence or absence of Tween-80. Significance is shown when *p*<0.05 (*). The *ppe10*::*tn* strain had significantly fewer bacteria per macrophage compared to wild-type *M*. *marinum* in the absence of detergent. The amount of bacteria per macrophage was highly dependent on the presence of Tween-80 for the *espG*_*5*_::*tn* strain in contrast to the *eccCb*_*1*_::*tn* strain which was always present in high numbers per macrophage.
(TIF)

S8 Fig The *ppe10*::*tn* mutant is able to translocate from the phagosome at late timepoints of infection. THP-1 cells infected with *M*. *marinum* wild-type, or *ppe10*::*tn* 48 hours after infection. Bacteria localize in the phagolysosome (A,B) and in the cytosol (C,D) of THP1 cells. Representative electron micrograph of CD63 labeled THP1 cell infected with E11 for 48 hours (A and C), and with *M*. *marinum ppe10*::*tn* strain for 48 hours (B and D). Mycobacteria were detected surrounded by phagosomal membrane (indicated M in white) and as cytosolic bacteria (indicated with M in black). Bar indicates magnification, L represents lysosomes and N nucleus. Quantification of 30 infected cells (containing more than 200 bacteria) per treatment, showed that 30% of the WT bacteria and 40% of the *ppe10*::*tn* mutant bacteria were present in the cytosol.
(TIF)

S1 Movie Ubiquitin co-localization of *M*. *marinum* infecting a macrophage. Wild type *M*. *marinum E11* expressing *mCherry* (Red) was used to infect THP-1 cells and cells were stained with the FK-2 antibody (Green) as described in Fig 4D. Images were obtained by STED super-resolution confocal microscopy and Z-stacks were combined using Huygens Professional Software (SVI). The movie show a three-dimensional view of the analyzed bacterial clusters and the threshold of green fluorescence was varied to visualize staining intensity.
(AVI)

S2 Movie The *ppe10*::*tn* mutant has reduced co-localization with ubiquitin and a single cell phenotype in cell-infection. The *ppe10*::*tn* mutant (Supplemental Movie S2) expressing *mCherry* (Red) was used to infect THP-1 cells and cells were stained and analyzed as described above. Compared to wild-type *M*. *marinum*, the *ppe10*::*tn* mutant showed less labelling with the FK2 antibody and showed a single-cell phenotype.
(AVI)
