# Supplementary figures and images for: The ESX-5 System of Pathogenic Mycobacteria Is Involved In Capsule Integrity and Virulence through Its Substrate PPE10
Source: PLoS Pathog. 2016 Jun 9;12(6):e1005696. doi: 10.1371/journal.ppat.1005696 (PMC4900558; doi:10.1371/journal.ppat.1005696)

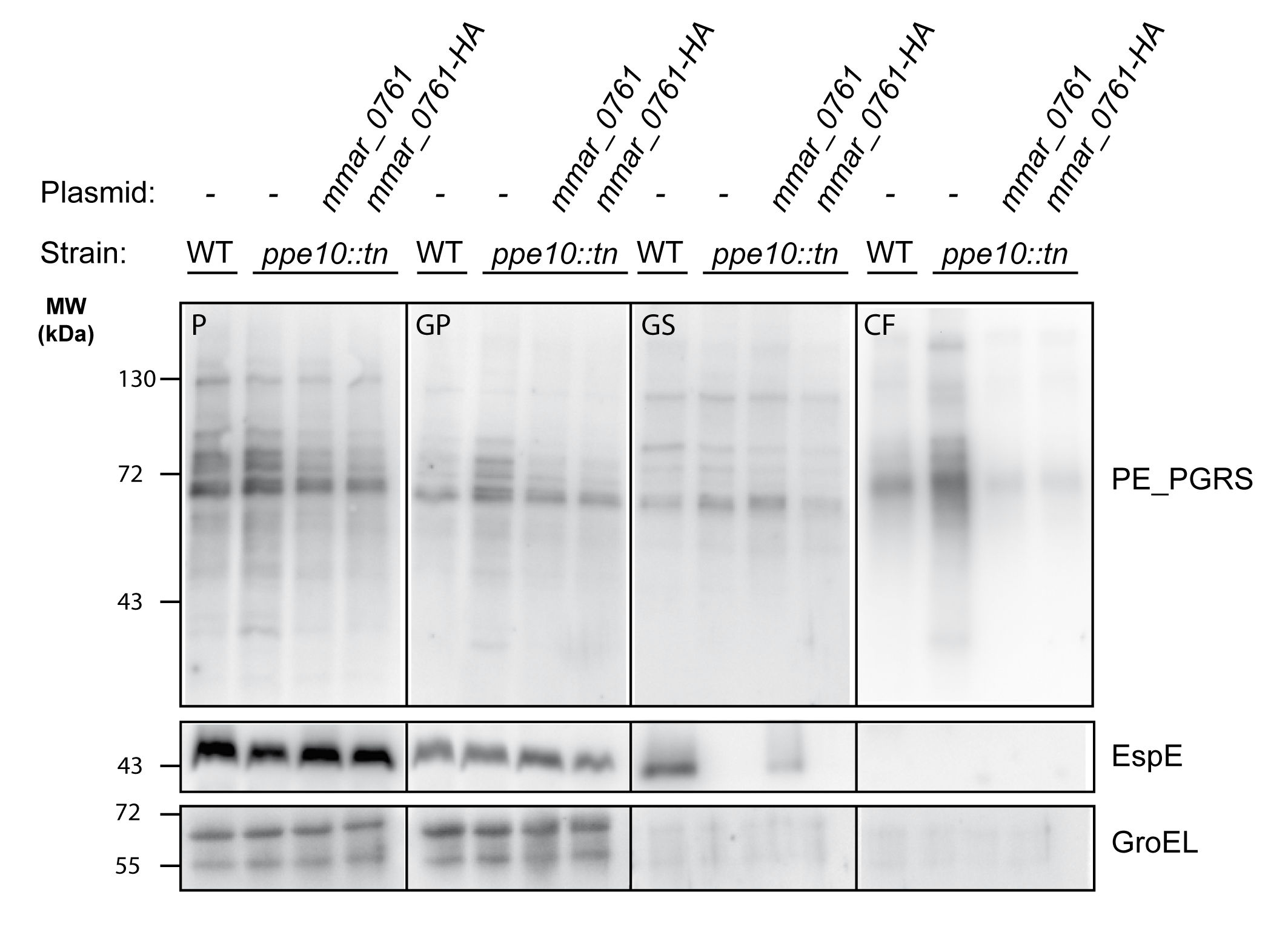

Supplement: S1 Fig — Immunoblot analysis of ppe10::tn complementation by overexpression of mmar_0761 on the episomal plasmid pSMT3 under the control of the hsp60 promoter. P = Whole cell lysate, GP = Genapol X-080 treated cells, GS = Supernatant of Genapol X-080 treated cells, CF = Culture filtrate. Complementation was performed with or without a C-terminal HA-tag fused to mmar_0761. The PE_PGRS supersecretor phenotype of ppe10::tn was restored upon introduction of native- or HA-tagged mmar_0761. However, surface localization of EspE was not restored with HA-tagged mmar_0761-HA, but was partially restored by mmar_0761. (TIF) [file ppat.1005696.s001.tif]

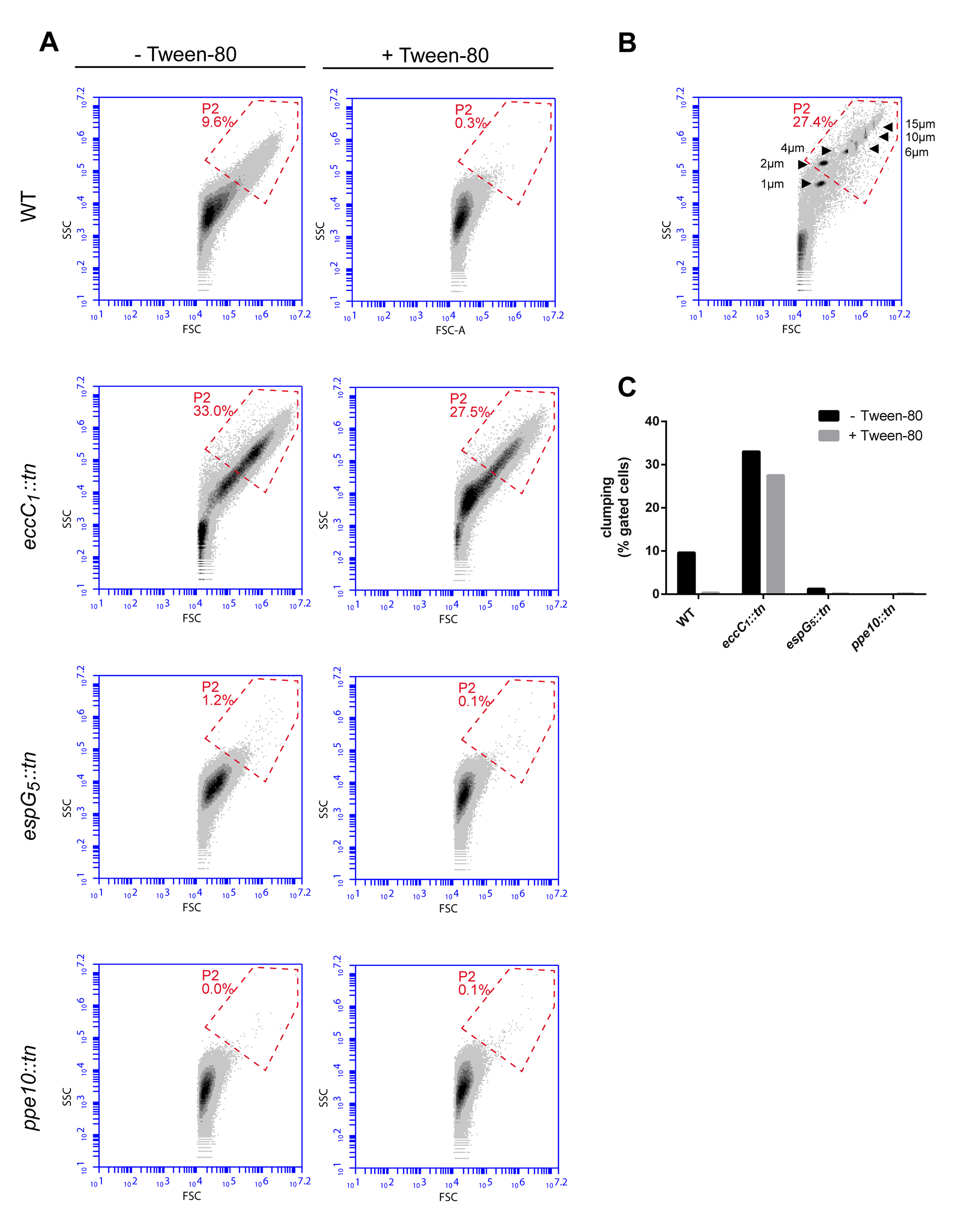

Supplement: S2 Fig — A) Flow cytometry plots showing forward scatter (FSC—x-axis) and side scatter (SSC—y-axis) of indicated M. marinum mutants in the presence or absence of 0.05% Tween-80. Gate P2 was set as a measurement of mycobacterial ‘clumping’, with the percentage of gated cells depicted in red. The presence of Tween-80 reduces clumping in all mutants. However, espG 5::tn and ppe10::tn show considerably less clumping compared to the wild-type, while the eccCb 1::tn shows larger aggregates. B) Size estimation of analyzed particles. Black arrows indicate sizes of calibration beads used as a reference for particle sizes. C) Quantification of gated cells of plots depicted in [A]. (TIF) [file ppat.1005696.s002.tif]

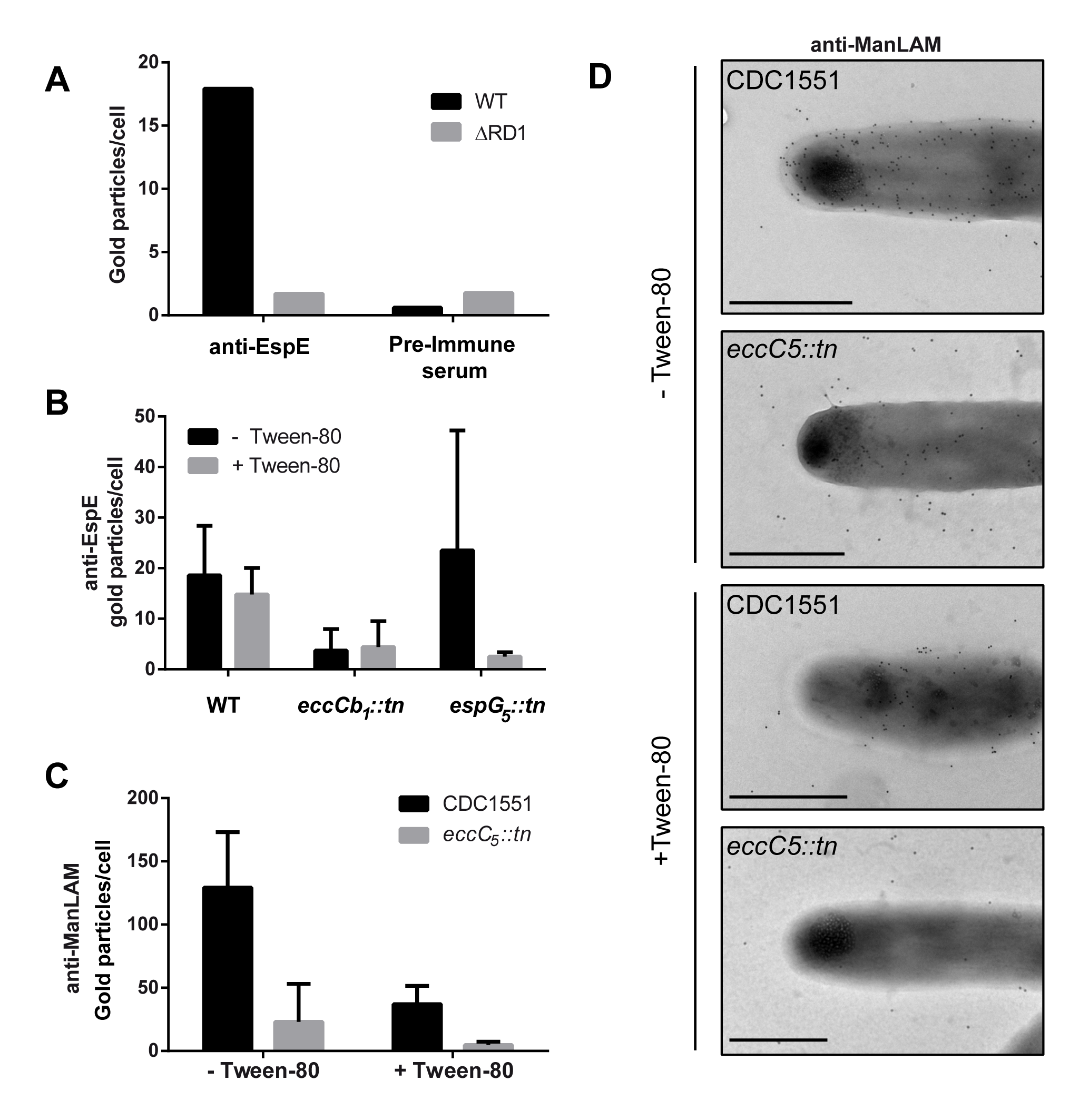

Supplement: S3 Fig — A) Quality and specificity control of the anti-EspE polyclonal antibody. M. marinum MUSA or the RD1 deletion strain Δmmar_3871–9 [32] (ΔRD1) were labeled by the anti-EspE serum or by pre-immune rabbit serum followed by a gold-labeled secondary antibody. Specific surface labeling was only observed in the wild-type bacteria labeled with the anti-EspE serum, indicating that this antibody specifically labels EspE. B) Quantification of EspE surface labeling of M. marinum strains. EspE surface labeling could be detected in wild-type M. marinum irrespective of the presence of Tween-80. EspE surface labeling was reduced to levels of the negative control eccCb 1::tn when espG 5::tn was grown with Tween-80. C) Quantification of electron microscopy surface labeling of M. tuberculosis wild-type strain CDC1551 or an isogenic ESX-5 mutant strain eccC 5::tn by an anti-Mannose-capped-lipoarabinomannan (ManLAM) antibody. Surface labeling of ManLAM is reduced in CDC1551 in the presence of Tween-80 and is markedly less in the eccC 5::tn strain irrespective of the presence of Tween-80. D) Transmission electron microscopy images of representative images from the dataset depicted in C. The length of the black scale bars represents 500 nm. (TIF) [file ppat.1005696.s003.tif]

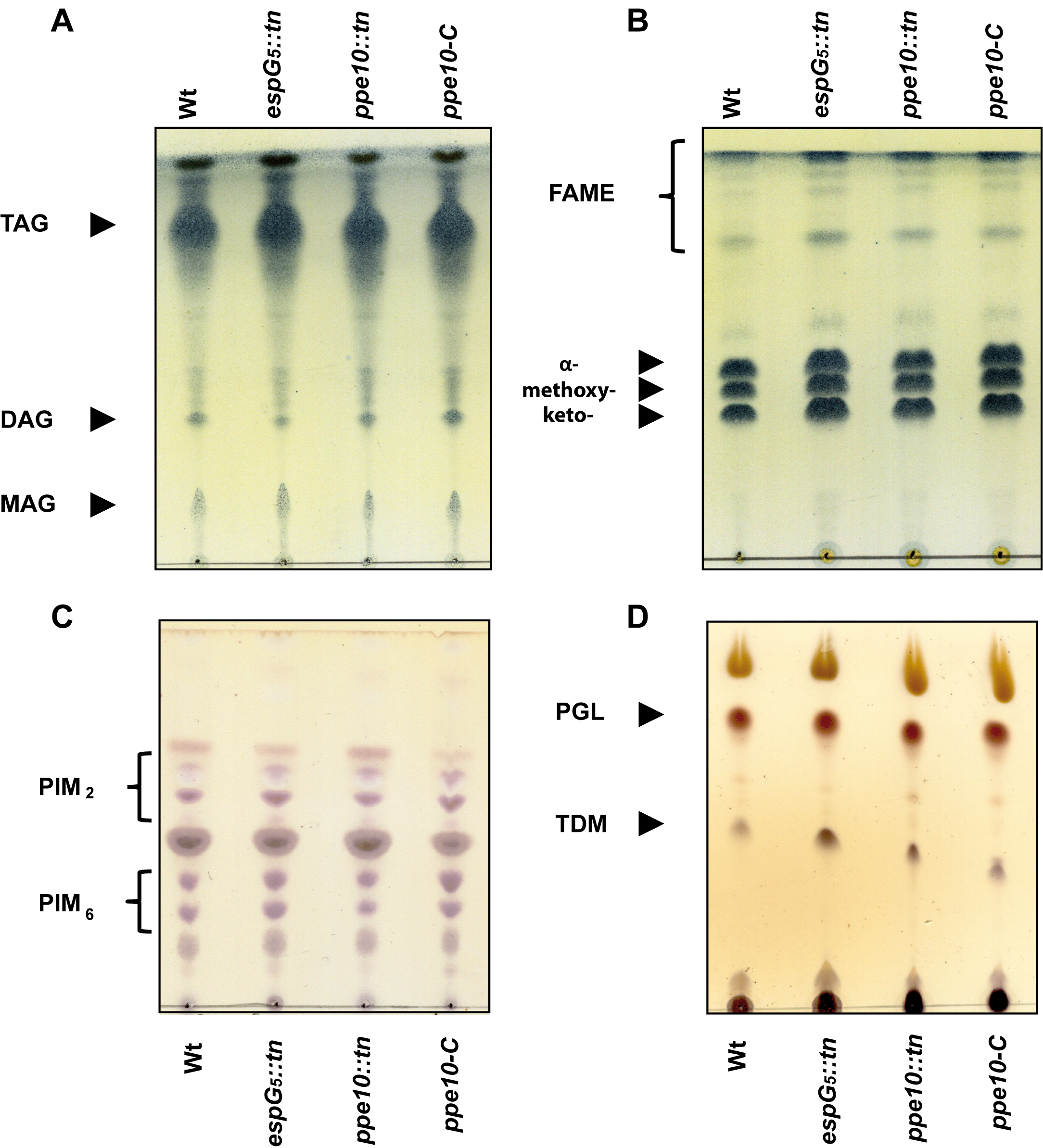

Supplement: S4 Fig — A) Apolar lipids fractions were separated by TLC using heptane/di-isopropyl ether/acetic acid (60:40:3, v/v/v) solvent and analyzed for the acyl-glycerol classes. The arrows indicate mono- (MAG), di- (DAG) and tri- (TAG) acyl-glycerols respectively. B) Mycolic acid fractions were separated by TLC using hexane/ethyl acetate (19:1, v/v) solvent. Arrows indicate the α-, methoxy- and keto- forms of the mycolic acids respectively, as well as the fatty acid methyl esters (FAMEs). C) Polar lipids separated by 1D-TLC using chloroform/acetic acid/methanol/water (40:25:3:6, v/v/v/v) solvent. Phosphatidylinositol mannosides (PIM) containing 2 (PIM2) and 6 (PIM6) mannose residues are respectively depicted. D) 1D-TLC of apolar lipids separated by chloroform/methanol (90:10, v/v). Arrows indicate phenolic glycolipids (PGL) and trehalose dimycolate (TDM). The lipids were visualized by 5% MPA in ethanol (A and B) or 5% orginol in 20% H2SO4 (C and D) and subsequent plate charring. PPE10-C = ppe10::tn expressing pSMT3::mmar_0761. (TIF) [file ppat.1005696.s004.tif]

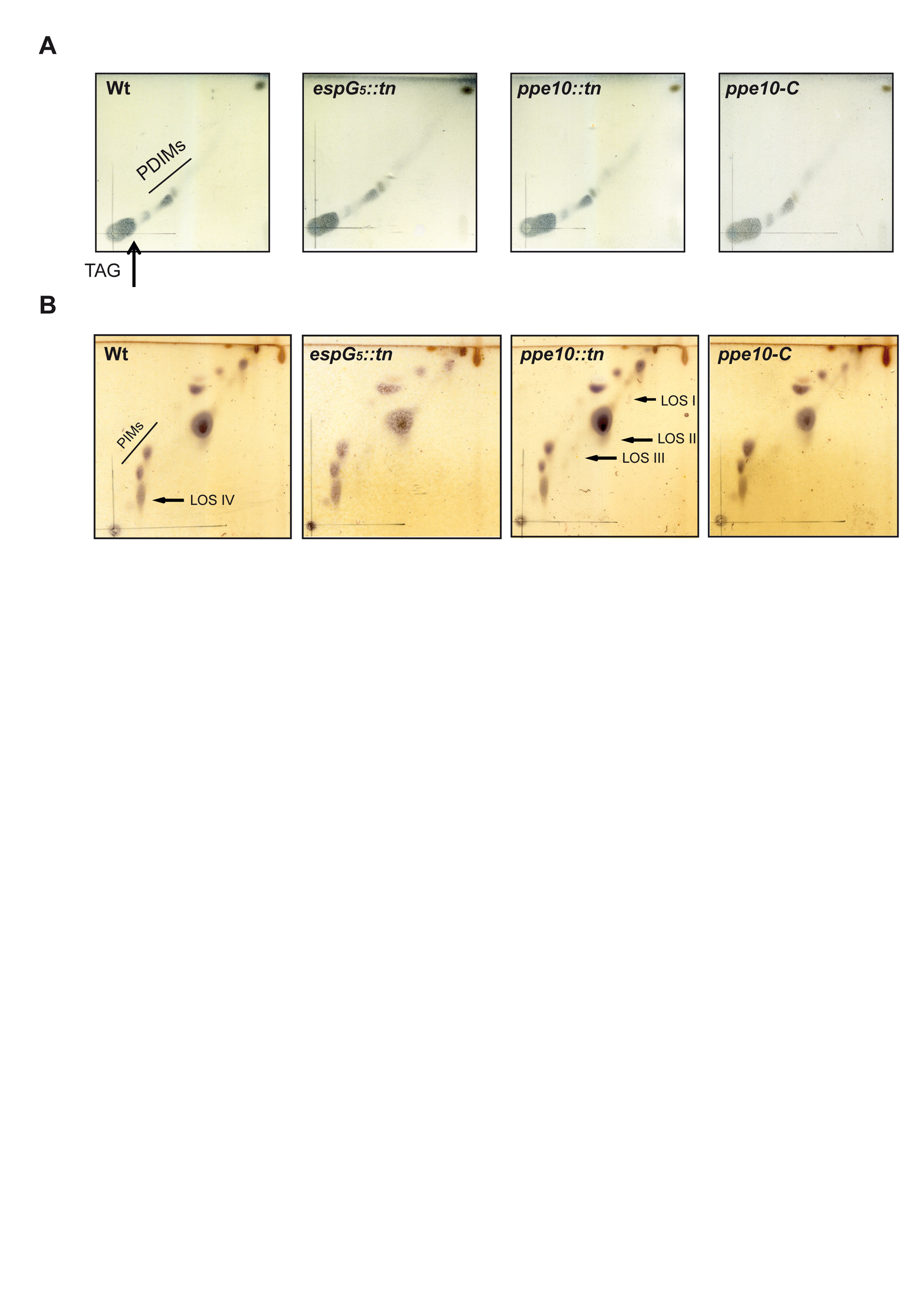

Supplement: S5 Fig — A) Analysis of PDIM lipids. Apolar lipids were separated by 2D-TLC with petroleum ether/ethyl acetate (98:2 v/v) and petroleum ether/ acetone (98:2 v/v) solvents respectively and were visualized by spraying with 5% MPA and plate charring. Location of PDIMs and TAGs are indicated by black arrows. B) 2D-TLC analysis of LOS and PIM glycolipids. Polar lipids were separated by TLC using chloroform/methanol/water (20:10:2, v/v/v) and chloroform/acetic acid/methanol/water (40:25:3:6, v/v/v/v) solvents respectively and were visualized by orginol spraying and plate charring. PIMs and different LOS fractions are indicated with the black line and arrows respectively. ppe10-C = ppe10::tn expressing pSMT3::mmar_0761. (TIF) [file ppat.1005696.s005.tif]

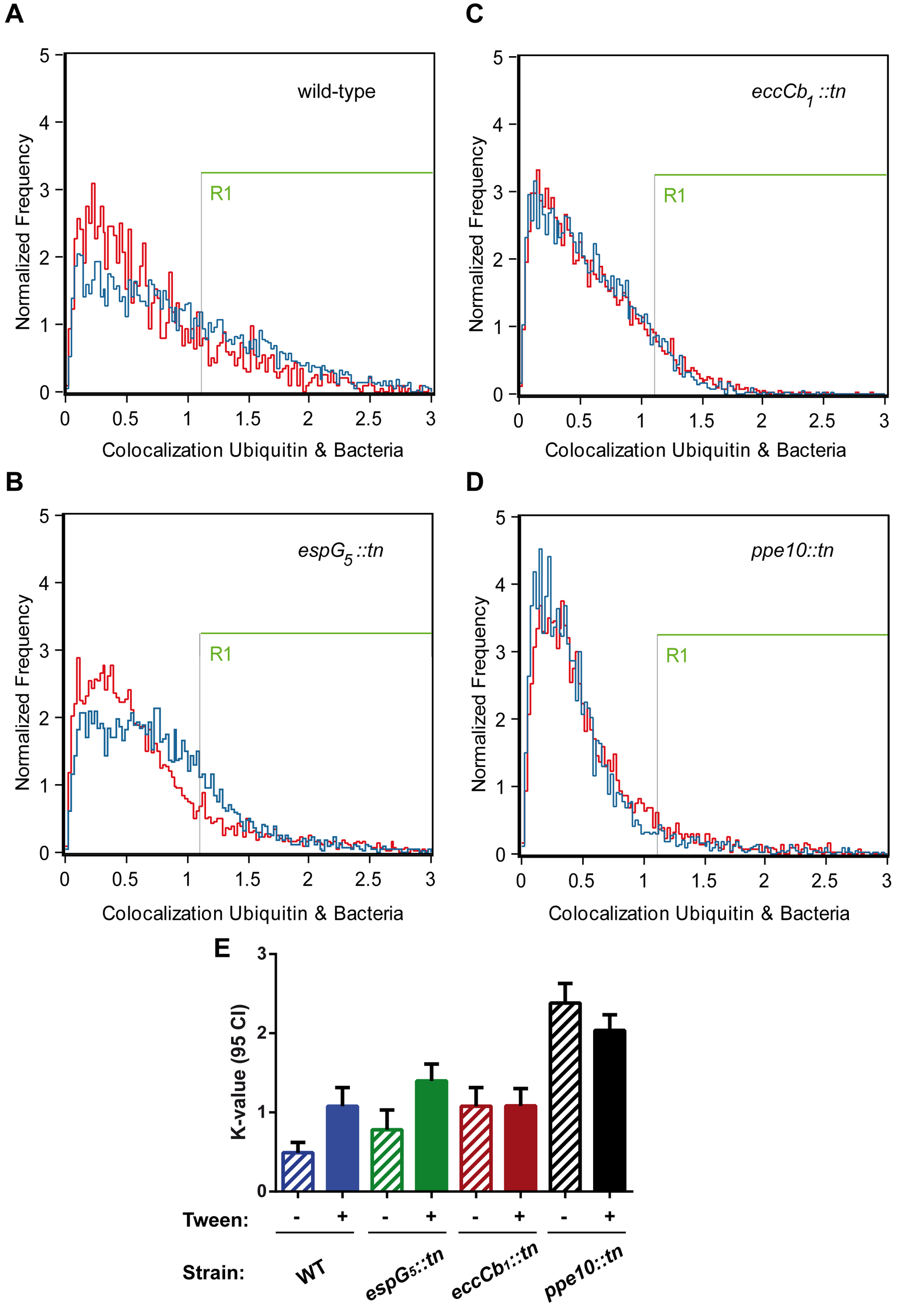

Supplement: S6 Fig — A-D) THP-1 macrophages infected with the indicated strain of M. marinum expressing mCherry were stained with the FK2 antibody recognizing poly-ubiquitin and were analyzed by imaging flow cytometry. Bacteria were pre-cultured in the presence (Red lines) or absence (Blue lines) of Tween-80. Relative co-localization of green and red fluorescence was quantified per particle (X-axis). Cells within gate R1 (green line) were seen as positive for co-localization of ubiquitin and bacteria for further analyses. Data of two independent experiments were pooled and analyzed together. E) The fluorescence intensity data depicted in the histogram plots (S6A–S6D Fig) was fitted to a one phase decay (Y = (Y0—Plateau)*exp(-K*X) + Plateau) with the constrain of the plateau set to 0. The goodness of fit for all data sets was greater than 0.9. The rate constant (K) was plotted and 95% CI intervals are shown. Non-overlapping confidence intervals are necessarily significantly different. The significantly higher rate constant for wild-type (Blue) and espG 5::tn (Green) in the presence of Tween, indicate fewer ubiquitin associated bacteria. The highest K value (lowest amount of ubiquitin associated bacteria) was observed for the ppe10::tn mutant (Black), which was independent for the presence (Filled bars) or absence (Striped bars) of Tween. (TIF) [file ppat.1005696.s006.tif]

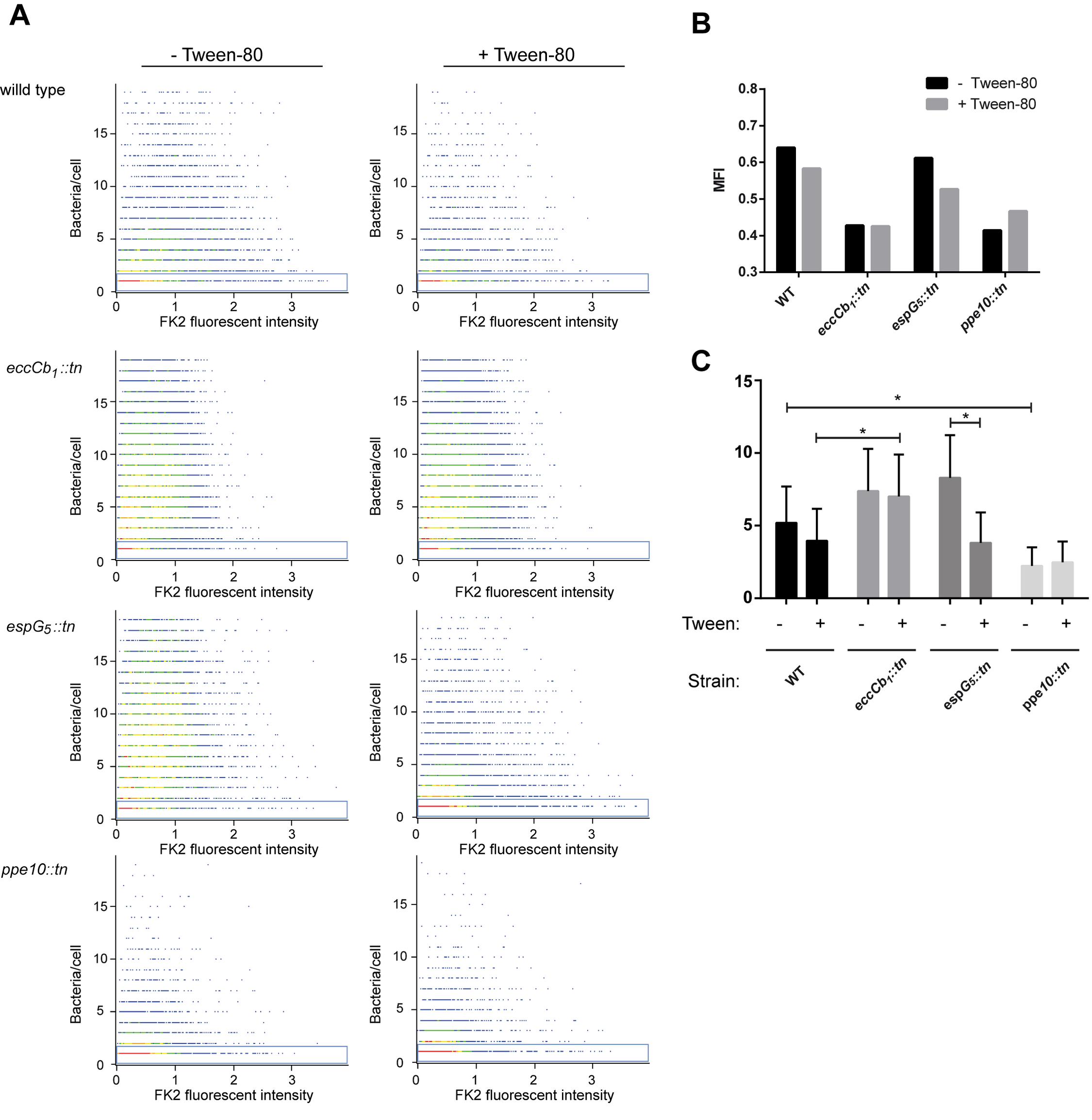

Supplement: S7 Fig — THP-1 macrophages were infected by the indicated strains of M. marinum expressing mCherry and were pre-cultured in the presence or absence of Tween-80. Infected cells were stained with the FK2 antibody and a FITC-labeled secondary antibody. A) Cells were analyzed by imaging flow cytometry and were sorted for the amount of bacteria per cell (Y-axis) and the intensity of FK2 staining (X-axis). Color coding indicates the density of analyzed particles with indicated fluorescent intensities. The blue rectangle indicates the gate used to analyze macrophages infected by a single bacterium. B) Analysis of the mean fluorescent intensity (MFI) of FK-2 staining measured by the FITC signal (Y-axis) of gated cells that contain only a single bacterium. Relative MFI data correlated very well with data obtained in other assays and analyses (Fig 4A and 4B) indicating that the number of bacteria per macrophage is not the driving force behind the observed phenotype of ubiquitin co-localization. C) Average number of bacteria per infected macrophage. SEM values are shown from three independent experiments. The Kolmogorov-Smirnov test (data is not Gaussian distributed) was used to determine statistical significance between identical strains and between wild type and mutant strain grown in the presence or absence of Tween-80. Significance is shown when p<0.05 (*). The ppe10::tn strain had significantly fewer bacteria per macrophage compared to wild-type M. marinum in the absence of detergent. The amount of bacteria per macrophage was highly dependent on the presence of Tween-80 for the espG 5::tn strain in contrast to the eccCb 1::tn strain which was always present in high numbers per macrophage. (TIF) [file ppat.1005696.s007.tif]

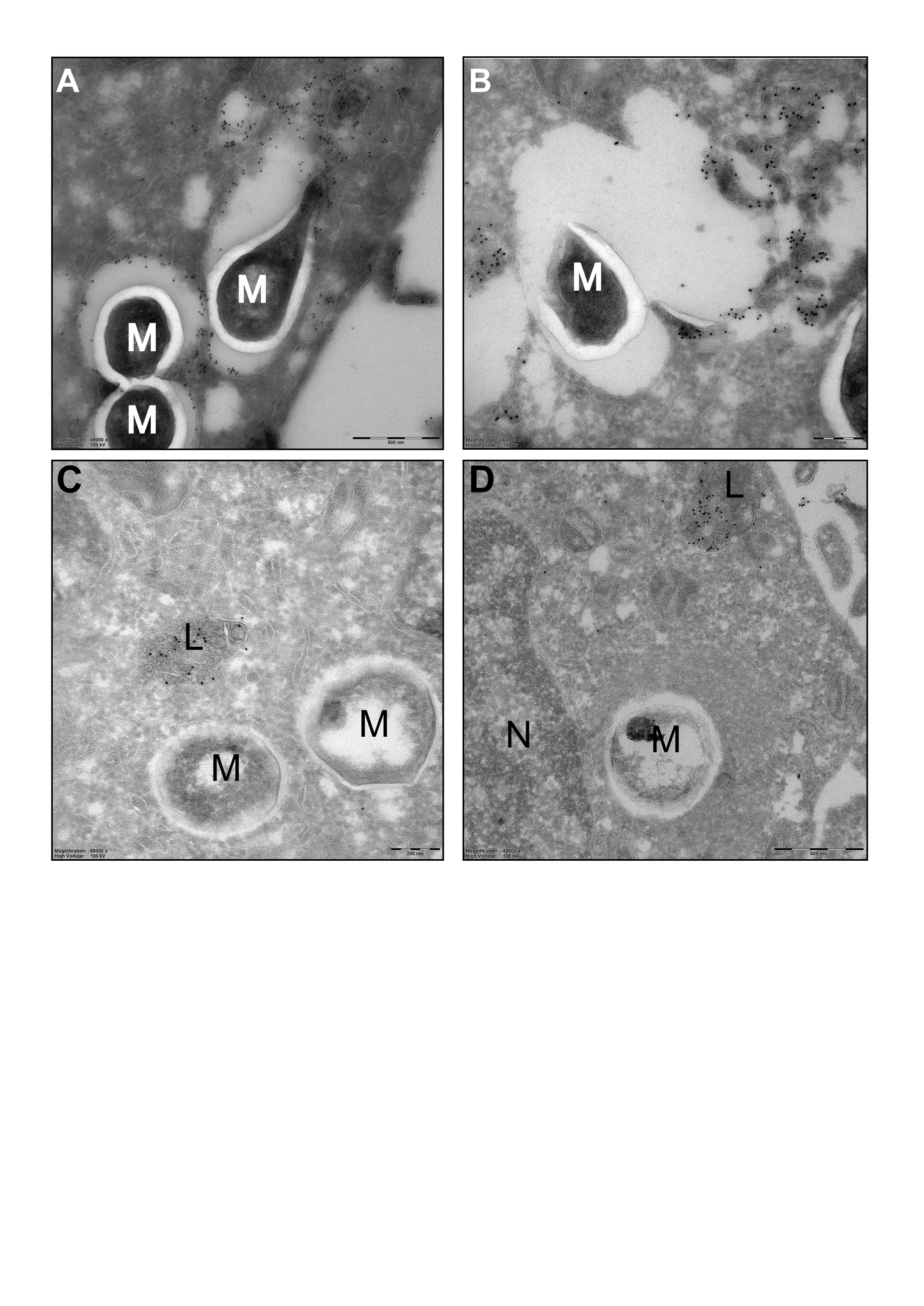

Supplement: S8 Fig — THP-1 cells infected with M. marinum wild-type, or ppe10::tn 48 hours after infection. Bacteria localize in the phagolysosome (A,B) and in the cytosol (C,D) of THP1 cells. Representative electron micrograph of CD63 labeled THP1 cell infected with E11 for 48 hours (A and C), and with M. marinum ppe10::tn strain for 48 hours (B and D). Mycobacteria were detected surrounded by phagosomal membrane (indicated M in white) and as cytosolic bacteria (indicated with M in black). Bar indicates magnification, L represents lysosomes and N nucleus. Quantification of 30 infected cells (containing more than 200 bacteria) per treatment, showed that 30% of the WT bacteria and 40% of the ppe10::tn mutant bacteria were present in the cytosol. (TIF) [file ppat.1005696.s008.tif]
